# Supplementary material for: The evolution of environmentally mediated social interactions and posthumous spite under isolation by distance
Source: PLoS Comput Biol. 2024 May 30;20(5):e1012071. doi: 10.1371/journal.pcbi.1012071 (PMC11139344; doi:10.1371/journal.pcbi.1012071)
Supplement: S1 Text — (PDF) [file pcbi.1012071.s001.pdf]

S1 Text

Appendix A Convergence stability from fixation probability

Here, we prove eqs. (4)-(6) of the main text by considering the fixation probability of a single mutant with trait value $z+\delta$ into a population monomorphic for resident trait z. Let $\Pi(z+\delta, z)$ denote the fixation probability of this mutant, and by

$$\phi(z) = \left. \frac{\mathrm{d}\Pi(z+\delta, z)}{\mathrm{d}\delta} \right|_{\delta=0} \tag{A-1}$$

the derivative of the fixation probability with respect to the mutant effect. A trait value z^* that is convergence stable under a trait substitution sequence is thus characterized by

$$\phi(z^*) = 0 \qquad \text{and} \qquad \left. \frac{\mathrm{d}\phi(z)}{\mathrm{d}z} \right|_{z=z^*} < 0 \tag{A-2}$$

[1, 2, 3]. Under our modeling assumptions, the perturbation of the fixation probability is given by

$$\phi(z) = \lim_{\mu \to 0} \left(\frac{1-\overline{Q}_0}{1-Q_{\mathbf{0},0}} \right) \times \left(\frac{\partial w(z_\bullet, \mathbf{z}_{\mathbf{0},0}, \mathbf{n}_{\mathbf{0},0}(\mathbf{z}_\mathrm{H}))}{\partial z_\bullet} + \sum_{t=0}^{\infty} \sum_{\mathbf{k} \in \mathrm{G}} \frac{\partial w(z_\bullet, \mathbf{z}_{\mathbf{0},0}, \mathbf{n}_{\mathbf{0},0}(\mathbf{z}_\mathrm{H}))}{\partial z_{\mathbf{k},t}} R_{\mathbf{k},t} \right) \tag{A-3}$$

(eq. 1 of [4] together with eq. A11 of [5]), which can be expressed as

$$\phi(z) = \underbrace{\lim_{\mu \to 0} \left(\frac{1-\overline{Q}_0}{1-Q_{\mathbf{0},0}} \right)}_{>0} s(z), \tag{A-4}$$

where $s(z)$ is given by eqs. (5)–(6b). Because the limit in eq. (A-4) is always positive as long as $N > 1$ holds [2], the condition for convergence stability (A-2) is equivalently given by eq. (4).

The condition for convergence stability (A-2) also connects to the stationary probability density function $p(z)$ that trait value z is observed in the population under a trait substitution sequence process in a finite population. This probability density function is given by

$$p(z) = K \exp\left[2DN \int_l^z \phi(y)\,\mathrm{d}y \right], \tag{A-5}$$

(eq. 7. of [6], eq. 62 of [3]) where l is the lower boundary of the state space and $p(z)$ has a local maximum at z^* if conditions (A-2) are satisfied (see e.g. [6, 3] for details). The density function (A-5) is useful to evaluate the expected phenotypic variance in the population and can thus be compared to results from individual-based simulations (see eq. A-136 and Fig 6B for a concrete example).

To compute the probability density function $p(z)$, however, requires to fully quantify the derivative of the fixation probability $\phi(z)$, which in turn depends on $\lim_{\mu\to 0}\left(1-\overline{Q}_0\right)/\left(1-Q_{0,0}\right)$, which is process specific. For instance, for the Wright-Fisher process,

$$\lim_{\mu\to 0}\left(\frac{1-\overline{Q}_0}{1-Q_{0,0}}\right)=\left(\frac{DN+M}{DN}\right) \tag{A-6}$$

holds (eq. A17 in [4]), where $M=\sum_{\mathbf{h}\in\mathbb{G}\setminus\mathbf{0}}\mathcal{M}(\mathbf{h})^2/\left(1-\mathcal{M}(\mathbf{h})^2\right)$ is defined as under eq. (8) in the main text and may remain complicated to evaluate. Yet evaluating eq. (A-6) may actually not be needed to compute $\phi(z)$. For instance when the selection gradient takes the form of eq. (A-55) (eq. (19) of the main text), eq. (A-6) cancels from $\phi(z)$ when fitness takes the form eq. (17) owing to eq. (A-105) (and this property may hold more generally). Further, using coalescent arguments, eq. (A-6) may be computed indirectly. For instance, owing to eq. (3.68) and eq. (3.70) of [2], one actually has $\lim_{\mu\to 0}\left[(1-\overline{Q}_0)/(1-Q_{0,0})\right]=1/(DN)$ for the Wright-Fisher process under the infinite allele model.

Appendix B A distribution for short and long range dispersal

Here, we specify a dispersal distribution based on the binomial distribution, which allows us to consider both short and long dispersal, and that we used to generate the various numerical examples of our analysis.

Appendix B.1 One-dimensional habitat

Let us first consider a one-dimensional habitat consisting of a circular lattice, so that the set of patches is $\mathbb{G}=\mathbb{Z}_D=\{0,1,\ldots,D-1\}$, i.e. the set of integers modulo D. We assume that D is odd, so that we can write $\mathbb{Z}_D=\{0,1,\ldots,(D-1)/2,-(D-1)/2,-(D-1)/2+1,\ldots,-1\}$. We further assume that an individual disperses with probability m, and that it stays in its natal patch with probability $1-m$. If an individual disperses, it does so with equal probability either "clockwise" or "counterclockwise" a number $j\in\{1,2,...,(D-1)/2\}$ of steps, which we assume follows a zero-truncated binomial distribution with probability mass function

$$p_j(N_\mathrm{s},q)=\frac{\binom{N_\mathrm{s}}{j}q^j(1-q)^{N_\mathrm{s}-j}}{1-(1-q)^{N_\mathrm{s}}}. \tag{A-7}$$

Here, $N_\mathrm{s}=(D-1)/2$ is the number of trials, and $q=2\lambda_\mathrm{m}/(D-1)$ is the probability of success, where $\lambda_\mathrm{m}=N_\mathrm{s}q$ is the mean of the non-truncated distribution. The mean number of steps an individual

disperses conditional on dispersal is given by

$$\overline{\lambda}_{\mathrm{m}} = \frac{\lambda_{\mathrm{m}}}{1 - \left(1 - \frac{2\lambda_{\mathrm{m}}}{D-1}\right)^{(D-1)/2}}. \tag{A-8}$$

From these assumptions, the dispersal distribution is given by

$$m_j = m_{-j} = \begin{cases} 1 - m, & \text{if } j = 0 \\ \frac{1}{2} m p_j(N_{\mathrm{s}}, q) & \text{if } j \in \{1, 2, ..., (D-1)/2\}, \end{cases} \tag{A-9}$$

and its associated characteristic function (or Fourier transform I.B) can be written as

$$\begin{aligned} \mathcal{M}(k) &= \sum_{j=0}^{D-1} m_j \chi_j(k) \\ &= m_0 + \sum_{j=1}^{\frac{D-1}{2}} m_j \chi_j(k) + \sum_{j=1}^{\frac{D-1}{2}} m_{-j} \chi_{-j}(k) \\ &= m_0 + \sum_{j=1}^{\frac{D-1}{2}} m_j \left(\chi_j(k) + \overline{\chi}_j(k)\right) \\ &= (1-m) + m \sum_{j=1}^{\frac{D-1}{2}} p_j((D-1)/2, 2\lambda_{\mathrm{m}}/(D-1)) \left(\frac{\chi_j(k) + \overline{\chi}_j(k)}{2}\right) \\ &= (1-m) + m \sum_{j=1}^{\frac{D-1}{2}} p_j((D-1)/2, 2\lambda_{\mathrm{m}}/(D-1)) \cos\left(2\pi jk/D\right), \end{aligned} \tag{A-10}$$

where the third line uses the fact that the migration kernel is symmetric ($m_j = m_{-j}$ holds for $j \in \{1, 2, ..., (D-1)/2\}$) and the identity $\chi_{-j}(k) = \overline{\chi}_j(k)$, and the last line uses the trigonometric identity $\cos(x) = (\exp(\iota x) + \exp(-\iota x))/2$. Eq. (A-10) shows that the characteristic function of the dispersal distribution is determined by the parameters D, m, and λ_{m}.

Appendix B.2 Two-dimensional habitat

For the two-dimensional case, we consider a torus with the same number of patches in each dimension so that $\mathbb{G} = \{(k_1, k_2) : 0 \leq k_j < D^{1/2}\}$ for k_1 and k_2 modulo $D^{1/2}$. The dispersal distribution of the focal species $m_{\mathbf{k}}$ for $\mathbf{k} = (k_1, k_2) \in \mathbb{G}$, is constructed similarly as above. First, an individual disperses with probability m and with probability $1 - m$ stays in its natal patch. Second, conditional on dispersal, we sample the number of steps $j \in \{1, 2, ..., D^{1/2} - 1\}$ an individual disperses on the lattice (maximum $D^{1/2} - 1$) from a zero-truncated binomial distribution $p_j(N_{\mathrm{s}}, q)$ (eq. A-7) with parameters $N_{\mathrm{s}} = D^{1/2} - 1$ and $q = \lambda_{\mathrm{m}}/(D^{1/2} - 1)$. Accordingly, the mean number of steps an individual disperses

conditional on dispersal is

$$\overline{\lambda}_{\mathrm{m}} = \frac{\lambda_{\mathrm{m}}}{1 - \left(1 - \frac{\lambda_{\mathrm{m}}}{D^{1/2}-1}\right)^{D^{1/2}-1}}. \tag{A-11}$$

Third, we determine how this total number of steps j is divided between j_1 steps in dimension 1 and j_2 steps in dimension 2 (so that $j = j_1 + j_2$), assuming that dispersal in either dimension has the same distribution. We do so by sampling j_1 from a discrete uniform distribution unif$(j_{\min}, j_{\max})$, where

$$\begin{aligned} j_{\min} &= \max\left(0, j - \frac{D^{1/2}-1}{2}\right) \\ j_{\max} &= \min\left(j, \frac{D^{1/2}-1}{2}\right), \end{aligned} \tag{A-12}$$

and by setting $j_2 = j - j_1$. Finally, given the number of steps in each dimension j_1 and j_2, these are then equally likely to occur in either direction away from the focal patch.

Appendix C Extended phenotypic effects

Appendix C.1 Actor-centered representation of inter-temporal effects

Here, we derive eq. (11) of the main text. To this end, we first apply the chain rule to the fitness expression (1) whereby we have for $t \geq 1$ that

$$\frac{\partial w(z_{\bullet}, \mathbf{z}_{\mathbf{0},0}, \mathbf{n}_{\mathbf{0},0}(\mathbf{z}_{\mathrm{H}}))}{\partial z_{\mathbf{k},t}} = \sum_{\mathbf{j}\in G}\left(N\frac{\partial w(z_{\bullet}, \mathbf{z}_{\mathbf{0},0}, \mathbf{n}_{\mathbf{0},0})}{\partial n_{\mathbf{j},0}}\right) e_{\mathbf{j}-\mathbf{k},t}, \tag{A-13}$$

where we have defined

$$e_{\mathbf{j}-\mathbf{k},t} = \frac{1}{N}\frac{\partial n_{\mathbf{j},0}(\mathbf{z}_{\mathrm{H}})}{\partial z_{\mathbf{k},t}}. \tag{A-14}$$

Thanks to spatial homogeneity this is in turn equivalent to

$$e_{\mathbf{j}-\mathbf{k},t} = \frac{1}{N}\frac{\partial n_{\mathbf{j}-\mathbf{k},0}(\mathbf{z}_{\mathrm{H}})}{\partial z_{\mathbf{0},t}}. \tag{A-15}$$

The quantity $e_{\mathbf{j}-\mathbf{k},t}$ is the extended phenotypic effect of a single individual residing in the focal patch at t time steps in the past on the value of the environmental variable in patch $\mathbf{j}-\mathbf{k}$ in the present, where $\partial n_{\mathbf{j}-\mathbf{k},0}(\mathbf{z}_{\mathrm{H}})/\partial z_{\mathbf{0},t}$ is the effect of the whole set of individuals in the focal patch at t time steps in the past on the value that the environmental variable takes in patch $\mathbf{j}-\mathbf{k}$ in the present. But since the map g (eq. 2) does not depend on time (i.e. environmental dynamics are homogeneous in time), $e_{\mathbf{j}-\mathbf{k},t}$ is also the effect of a focal individual residing in the focal patch on the value that the environmental

variable takes in patch $\mathbf{j}-\mathbf{k}$ at t time steps in the future. We can thus write

$$e_{\mathbf{j}-\mathbf{k},t} = \frac{\partial n_{\mathbf{j}-\mathbf{k},t}}{\partial z_\bullet}, \tag{A-16}$$

where $n_{\mathbf{k},t}$ now stands for the value of the environmental variable in patch $\mathbf{k}$ at t steps in the future. Substituting eq. (A-16) into eq. (A-13), and this into eq. (6b), obtains eq. (11), as required.

Appendix C.2 Extended phenotypic effects

Here, we derive the expression for the extended phenotypic effect given by eq. (14) of the main text. To do this, we first take the derivative on both sides of eq. (9) with respect to $z_\bullet$, which yields

$$\frac{\partial n_{\mathbf{k},t+1}}{\partial z_\bullet} = \delta_{t,0}\frac{\partial g(\mathbf{z}^{\mathrm{R}}_{\mathbf{k},0}, \mathbf{n}_{\mathbf{k},0})}{\partial z_\bullet} + \sum_{\mathbf{i}\in\mathbb{G}} \frac{\partial g(\mathbf{z}^{\mathrm{R}}_{\mathbf{k},t}, \mathbf{n}_{\mathbf{k},t})}{\partial n_{\mathbf{i},t}} \frac{\partial n_{\mathbf{i},t}}{\partial z_\bullet}, \tag{A-17}$$

where $\delta_{t,0}$ is a Kronecker delta, and where we used $\partial g(\mathbf{z}_{\mathbf{k},t}, \mathbf{n}_{\mathbf{k},t})/\partial n_{\mathbf{i},t} = \partial g(\mathbf{z}^{\mathrm{R}}_{\mathbf{k},t}, \mathbf{n}_{\mathbf{k},t})/\partial n_{\mathbf{i},t}$, since all derivatives are evaluated at z and $\hat{n}$. This also entails that the derivatives of the transition map g are independent of time, which allows us to write

$$e_{\mathbf{k},t+1} = \delta_{t,0}\psi_{\mathbf{k}} + \sum_{\mathbf{i}\in\mathbb{G}} c_{\mathbf{k}-\mathbf{i}} e_{\mathbf{i},t}, \tag{A-18}$$

with

$$\psi_{\mathbf{k}} = \frac{\partial g(\mathbf{z}^{\mathrm{R}}_{\mathbf{k},0}, \mathbf{n}_{\mathbf{k},0})}{\partial z_\bullet} = \begin{cases} \dfrac{1}{N}\dfrac{\partial g(\mathbf{z}^{\mathrm{R}}_{\mathbf{0},0}, \mathbf{n}_{\mathbf{0},0})}{\partial z^{\mathrm{R}}_{\mathbf{0},0}} & \text{for } \mathbf{k}=\mathbf{0} \\[2ex] \dfrac{1}{N}\dfrac{\partial g(\mathbf{z}^{\mathrm{R}}_{\mathbf{0},0}, \mathbf{n}_{\mathbf{0},0})}{\partial z_{\mathbf{k},0}} & \text{otherwise,} \end{cases} \tag{A-19}$$

and

$$c_{\mathbf{k}-\mathbf{i}} = \frac{\partial g(\mathbf{z}^{\mathrm{R}}_{\mathbf{k},t}, \mathbf{n}_{\mathbf{k},t})}{\partial n_{\mathbf{i},t}} = \frac{\partial g(\mathbf{z}^{\mathrm{R}}_{\mathbf{k},0}, \mathbf{n}_{\mathbf{k},0})}{\partial n_{\mathbf{i},0}} = \frac{\partial g(\mathbf{z}^{\mathrm{R}}_{\mathbf{0},0}, \mathbf{n}_{\mathbf{0},0})}{\partial n_{\mathbf{k}-\mathbf{i},t}}, \tag{A-20}$$

where the second equality in equation (A-19) follows from spatial homogeneity and the chain rule of derivatives, the second equality in equation (A-20) follows from temporal homogeneity, and the last equality in equation (A-20) follows from spatial homogeneity. These expressions are useful in concrete applications since only $g(\mathbf{z}^{\mathrm{R}}_{\mathbf{0},0}, \mathbf{n}_{\mathbf{0},0})$ needs to be specified to evaluate $\psi_{\mathbf{k}}$ and $c_{\mathbf{k}}$ (see section 3.4).

We can solve eq. (A-18), using the Fourier transforms (see Box 1) $\mathcal{E}_t(\mathbf{h}) = \sum_{\mathbf{k}\in\mathbb{G}} e_{\mathbf{k},t}\chi_{\mathbf{k}}(\mathbf{h})$, $\mathcal{C}(\mathbf{h}) = \sum_{\mathbf{k}\in\mathbb{G}} c_{\mathbf{k}}\chi_{\mathbf{k}}(\mathbf{h})$ (Table 1) and $\Psi(\mathbf{h}) = \sum_{\mathbf{k}\in\mathbb{G}} \psi_{\mathbf{k}}\chi_{\mathbf{k}}(\mathbf{h})$ (Table 1). Using these expressions, from (A-18),

and noting that $\chi_{\mathbf{k}}(\mathbf{h}) = \chi_{\mathbf{k}-\mathbf{i}}(\mathbf{h})\chi_{\mathbf{i}}(\mathbf{h})$ holds, we have

$$\underbrace{\sum_{\mathbf{k}\in\mathbb{G}} e_{i,\mathbf{k},t+1}\chi_{\mathbf{k}}(\mathbf{h})}_{\mathcal{E}_{t+1}(\mathbf{h})} = \delta_{t,0} \underbrace{\sum_{\mathbf{k}\in\mathbb{G}} \psi_{\mathbf{k}}\chi_{\mathbf{k}}(\mathbf{h})}_{\Psi(\mathbf{h})} + \underbrace{\sum_{\mathbf{i}\in\mathbb{G}} e_{\mathbf{i},t}\chi_{\mathbf{i}}(\mathbf{h})}_{\mathcal{E}_t(\mathbf{h})} \underbrace{\sum_{\mathbf{k}\in\mathbb{G}} c_{\mathbf{k}-\mathbf{i}}\chi_{\mathbf{k}-\mathbf{i}}(\mathbf{h})}_{\mathcal{C}(\mathbf{h})}, \tag{A-21}$$

where the expression for $\mathcal{C}(\mathbf{h})$ holds by changing the dummy index of the sum. Thus, we obtain the recursion

$$\mathcal{E}_{t+1}(\mathbf{h}) = \delta_{t,0}\Psi(\mathbf{h}) + \mathcal{C}(\mathbf{h})\mathcal{E}_t(\mathbf{h}), \tag{A-22}$$

whose solution given the initial condition $\mathcal{E}_0(\mathbf{h}) = 0$ (as there are no extended phenotypic effects in the focal generation) is $\mathcal{E}_t(\mathbf{h}) = \mathcal{C}(\mathbf{h})^{t-1}\Psi(\mathbf{h})$, as required in eq. (14).

Appendix D Selection gradient in terms of scaled relatedness

Here, we derive eq. (18) which, recall, is premised on the fitness of the focal individual taking the form

$$w(z_\bullet, \mathbf{z}_{\mathbf{0},0}, \mathbf{n}_{\mathbf{0},0}) = \tilde{w}(\boldsymbol{\pi}(z_\bullet, \mathbf{z}_{\mathbf{0},0}, \mathbf{n}_{\mathbf{0},0})) \tag{A-23}$$

with payoff vector

$$\boldsymbol{\pi}(z_\bullet, \mathbf{z}_{\mathbf{0},0}, \mathbf{n}_{\mathbf{0},0}) = \left(\underbrace{\pi(z_\bullet, \mathbf{z}_{\mathbf{0},0}, \mathbf{n}_{\mathbf{0},0})}_{\pi_\bullet}, \underbrace{\pi(z_{\mathbf{0},0}, \mathbf{z}^{\mathrm{n}}_{\mathbf{0},0}, \mathbf{n}_{\mathbf{0},0})}_{\pi_{\mathbf{0}}}, \dots, \underbrace{\pi(z_{\mathbf{j},0}, \mathbf{z}^{\mathrm{R}}_{\mathbf{j},0}, \mathbf{n}_{\mathbf{j},0})}_{\pi_{\mathbf{j}}}, \dots \right). \tag{A-24}$$

Here, $\mathbf{z}^{\mathrm{n}}_{\mathbf{0},0}$ is equivalent to $\mathbf{z}_{\mathbf{0},0}$ except for the first entry which is given by

$$z^{\mathrm{n}}_{\mathbf{0},0} = \frac{1}{N-1}z_\bullet + \frac{N-2}{N-1}z_{\mathbf{0},0}, \tag{A-25}$$

(instead $z_{\mathbf{0},0}$ of in $\mathbf{z}_{\mathbf{0},0}$), and $\mathbf{z}^{\mathrm{R}}_{\mathbf{j},0}$ is equal to $\mathbf{z}_{\mathbf{j},0}$ except that the entry with component $z_{\mathbf{0},0}$ in this vector is replaced with

$$z^{\mathrm{R}}_{\mathbf{0},0} = \frac{1}{N}z_\bullet + \frac{N-1}{N}z_{\mathbf{0},0}, \tag{A-26}$$

that is, with the average phenotype in the patch $\mathbf{0}, 0$ including the focal individual.

To simplify the operation of taking derivatives of fitness with respect to phenotypes later, we first express the derivatives of the payoff $\pi_{\mathbf{j}}$ appearing in eq. (A-24) with respect to its various arguments in terms of the derivatives of the payoff to the focal individual. Applying the chain rule of derivatives

and evaluating the derivatives at the resident phenotype, we readily obtain the following,

$$\frac{\partial \pi_\bullet}{\partial z_\bullet} = \frac{\partial \pi(z_\bullet, \mathbf{z}_{\mathbf{0},0}, \mathbf{n}_{\mathbf{0},0})}{\partial z_\bullet}, \tag{A-27}$$

$$\frac{\partial \pi_\bullet}{\partial z_{\mathbf{0},0}} = \frac{\partial \pi(z_\bullet, \mathbf{z}_{\mathbf{0},0}, \mathbf{n}_{\mathbf{0},0})}{\partial z_{\mathbf{0},0}}, \tag{A-28}$$

$$\frac{\partial \pi_{\mathbf{0}}}{\partial z_\bullet} = \frac{1}{N-1}\frac{\partial \pi(z_\bullet, \mathbf{z}_{\mathbf{0},0}, \mathbf{n}_{\mathbf{0},0})}{\partial z_{\mathbf{0},0}}, \tag{A-29}$$

$$\frac{\partial \pi_{\mathbf{j}}}{\partial z_\bullet} = \frac{1}{N}\frac{\partial \pi(z_\bullet, \mathbf{z}_{\mathbf{0},0}, \mathbf{n}_{\mathbf{0},0})}{\partial z_{\mathbf{j},0}} \quad \text{for } \mathbf{j} \neq \bullet, \mathbf{0}, \tag{A-30}$$

$$\frac{\partial \pi_{\mathbf{0}}}{\partial z_{\mathbf{0},0}} = \frac{\partial \pi(z_\bullet, \mathbf{z}_{\mathbf{0},0}, \mathbf{n}_{\mathbf{0},0})}{\partial z_\bullet} + \left(\frac{N-2}{N-1}\right)\frac{\partial \pi(z_\bullet, \mathbf{z}_{\mathbf{0},0}, \mathbf{n}_{\mathbf{0},0})}{\partial z_{\mathbf{0},0}}, \tag{A-31}$$

$$\frac{\partial \pi_{\mathbf{j}}}{\partial z_{\mathbf{0},0}} = \left(\frac{N-1}{N}\right)\frac{\partial \pi(z_\bullet, \mathbf{z}_{\mathbf{0},0}, \mathbf{n}_{\mathbf{0},0})}{\partial z_{\mathbf{j},0}} \quad \text{for } \mathbf{j} \neq \bullet, \mathbf{0}, \tag{A-32}$$

$$\frac{\partial \pi_{\mathbf{j}}}{\partial z_{\mathbf{j},0}} = \frac{\partial \pi(z_\bullet, \mathbf{z}_{\mathbf{0},0}, \mathbf{n}_{\mathbf{0},0})}{\partial z_\bullet} + \frac{\partial \pi(z_\bullet, \mathbf{z}_{\mathbf{0},0}, \mathbf{n}_{\mathbf{0},0})}{\partial z_{\mathbf{0},0}} \quad \text{for } \mathbf{j} \neq \mathbf{0}, \tag{A-33}$$

$$\frac{\partial \pi_{\mathbf{k}}}{\partial z_{\mathbf{j},0}} = \frac{\partial \pi_{\mathbf{j}}}{\partial z_{\mathbf{k},0}} = \frac{\partial \pi_\bullet}{\partial z_{\mathbf{j}-\mathbf{k},0}} = \frac{\partial \pi_\bullet}{\partial z_{\mathbf{k}-\mathbf{j},0}} = \frac{\partial \pi(z_\bullet, \mathbf{z}_{\mathbf{0},0}, \mathbf{n}_{\mathbf{0},0})}{\partial z_{\mathbf{k}-\mathbf{j},0}} = \frac{\partial \pi(z_\bullet, \mathbf{z}_{\mathbf{0},0}, \mathbf{n}_{\mathbf{0},0})}{\partial z_{\mathbf{j}-\mathbf{k},0}} \quad \text{for } \mathbf{j} \neq \mathbf{0} \text{ and } \mathbf{k} \neq \bullet, \mathbf{j}, \tag{A-34}$$

where the equalities in the last expression all follow from our assumption of spatial homogeneity.

Similarly, for derivatives of payoffs with respect to environmental state variables, we have

$$\frac{\partial \pi_\bullet}{\partial n_{\mathbf{0},0}} = \frac{\partial \pi_{\mathbf{j}}}{\partial n_{\mathbf{j},0}} = \frac{\partial \pi(z_\bullet, \mathbf{z}_{\mathbf{0},0}, \mathbf{n}_{\mathbf{0},0})}{\partial n_{\mathbf{0},0}} \quad \text{for all } \mathbf{j} \in \mathbb{G}, \tag{A-35}$$

where the first and second equalities are consequences of spatial homogeneity, and

$$\frac{\partial \pi_{\mathbf{k}}}{\partial n_{\mathbf{j},0}} = \frac{\partial \pi_\bullet}{\partial n_{\mathbf{j}-\mathbf{k},0}} = \frac{\partial \pi(z_\bullet, \mathbf{z}_{\mathbf{0},0}, \mathbf{n}_{\mathbf{0},0})}{\partial n_{\mathbf{j}-\mathbf{k},0}} \quad \text{for } \mathbf{j} \neq \mathbf{0} \tag{A-36}$$

where the first equality is again a consequence of spatial homogeneity.

We can then write the derivatives of fitness that appear in the selection gradient (eqs. 6a–6b) in terms of the derivatives of the payoff to the focal individual (eqs. A-27–A-36) by applying the chain rule of derivatives to the right-hand side of eq. (A-23) and simplifying, as follows. First, the fitness derivative

with respect to the focal individual's phenotype can be written as

$$
\begin{aligned}
\frac{\partial w(z_\bullet, \mathbf{z_0}, \mathbf{n}_{\mathbf{0},0})}{\partial z_\bullet} &= \frac{\partial \tilde{w}(\boldsymbol{\pi}(z_\bullet, \mathbf{z}_{\mathbf{0},0}, \mathbf{n}_{\mathbf{0},0}))}{\partial z_\bullet} \\
&= \frac{\partial \tilde{w}}{\partial \pi_\bullet}\frac{\partial \pi_\bullet}{\partial z_\bullet} + \frac{\partial \tilde{w}}{\partial \pi_\mathbf{0}}\frac{\partial \pi_\mathbf{0}}{\partial z_\bullet} + \sum_{\mathbf{k}\in\mathbf{G}\setminus\mathbf{0}} \frac{\partial \tilde{w}}{\partial \pi_\mathbf{k}}\frac{\partial \pi_\mathbf{k}}{\partial z_\bullet} \\
&= \frac{\partial \tilde{w}}{\partial \pi_\bullet}\frac{\partial \pi(z_\bullet, \mathbf{z}_{\mathbf{0},0}, \mathbf{n}_{\mathbf{0},0})}{\partial z_\bullet} + \frac{\partial \tilde{w}}{\partial \pi_\mathbf{0}}\frac{1}{N-1}\frac{\partial \pi(z_\bullet, \mathbf{z}_{\mathbf{0},0}, \mathbf{n}_{\mathbf{0},0})}{\partial z_{\mathbf{0},0}} \\
&+ \sum_{\mathbf{k}\in\mathbf{G}\setminus\mathbf{0}} \frac{\partial \tilde{w}}{\partial \pi_\mathbf{k}}\frac{1}{N}\frac{\partial \pi(z_\bullet, \mathbf{z}_{\mathbf{0},0}, \mathbf{n}_{\mathbf{0},0})}{\partial z_{\mathbf{k},0}},
\end{aligned} \tag{A-37}
$$

where the first equality follows from taking the derivative to both sides of eq. (A-23); the second equality follows from applying the chain rule; and the third equality follows from substituting eqs. (A-27)–(A-30).

Second, the fitness derivative with respect to the average phenotype of patch neighbours is

$$
\begin{aligned}
\frac{\partial w(z_\bullet, \mathbf{z_0}, \mathbf{n}_{\mathbf{0},0})}{\partial z_{\mathbf{0},0}} &= \frac{\partial \tilde{w}(\boldsymbol{\pi}(z_\bullet, \mathbf{z}_{\mathbf{0},0}, \mathbf{n}_{\mathbf{0},0}))}{\partial z_{\mathbf{0},0}} \\
&= \frac{\partial \tilde{w}}{\partial \pi_\bullet}\frac{\partial \pi_\bullet}{\partial z_{\mathbf{0},0}} + \frac{\partial \tilde{w}}{\partial \pi_\mathbf{0}}\frac{\partial \pi_\mathbf{0}}{\partial z_{\mathbf{0},0}} + \sum_{\mathbf{k}\in\mathbf{G}\setminus\mathbf{0}} \frac{\partial \tilde{w}}{\partial \pi_\mathbf{k}}\frac{\partial \pi_\mathbf{k}}{\partial z_{\mathbf{0},0}} \\
&= \frac{\partial \tilde{w}}{\partial \pi_\bullet}\frac{\partial \pi(z_\bullet, \mathbf{z}_{\mathbf{0},0}, \mathbf{n}_{\mathbf{0},0})}{\partial z_{\mathbf{0},0}} + \frac{\partial \tilde{w}}{\partial \pi_0}\left[\frac{\partial \pi(z_\bullet, \mathbf{z}_{\mathbf{0},0}, \mathbf{n}_{\mathbf{0},0})}{\partial z_\bullet} + \left(\frac{N-2}{N-1}\right)\frac{\partial \pi(z_\bullet, \mathbf{z}_{\mathbf{0},0}, \mathbf{n}_{\mathbf{0},0})}{\partial z_{\mathbf{0},0}}\right] \\
&+ \sum_{\mathbf{k}\in\mathbf{G}\setminus\mathbf{0}} \frac{\partial \tilde{w}}{\partial \pi_\mathbf{k}}\left(\frac{N-1}{N}\right)\frac{\partial \pi(z_\bullet, \mathbf{z}_{\mathbf{0},0}, \mathbf{n}_{\mathbf{0},0})}{\partial z_{\mathbf{k},0}} \\
&= \frac{\partial \tilde{w}}{\partial \pi_\bullet}\frac{\partial \pi(z_\bullet, \mathbf{z}_{\mathbf{0},0}, \mathbf{n}_{\mathbf{0},0})}{\partial z_{\mathbf{0},0}} + \frac{\partial \tilde{w}}{\partial \pi_0}\left[\frac{\partial \pi(z_\bullet, \mathbf{z}_{\mathbf{0},0}, \mathbf{n}_{\mathbf{0},0})}{\partial z_\bullet} + \left(1-\frac{1}{N-1}\right)\frac{\partial \pi(z_\bullet, \mathbf{z}_{\mathbf{0},0}, \mathbf{n}_{\mathbf{0},0})}{\partial z_{\mathbf{0},0}}\right] \\
&+ \sum_{\mathbf{k}\in\mathbf{G}\setminus\mathbf{0}} \frac{\partial \tilde{w}}{\partial \pi_\mathbf{k}}\left(1-\frac{1}{N}\right)\frac{\partial \pi(z_\bullet, \mathbf{z}_{\mathbf{0},0}, \mathbf{n}_{\mathbf{0},0})}{\partial z_{\mathbf{k},0}} \\
&= \frac{\partial \tilde{w}}{\partial \pi_\bullet}\frac{\partial \pi(z_\bullet, \mathbf{z}_{\mathbf{0},0}, \mathbf{n}_{\mathbf{0},0})}{\partial z_{\mathbf{0},0}} + \frac{\partial \tilde{w}}{\partial \pi_0}\left[\frac{\partial \pi(z_\bullet, \mathbf{z}_{\mathbf{0},0}, \mathbf{n}_{\mathbf{0},0})}{\partial z_\bullet} - \left(\frac{1}{N-1}\right)\frac{\partial \pi(z_\bullet, \mathbf{z}_{\mathbf{0},0}, \mathbf{n}_{\mathbf{0},0})}{\partial z_{\mathbf{0},0}}\right] \\
&+ \sum_{\mathbf{k}\in\mathbf{G}} \frac{\partial \tilde{w}}{\partial \pi_\mathbf{k}}\frac{\partial \pi(z_\bullet, \mathbf{z}_{\mathbf{0},0}, \mathbf{n}_{\mathbf{0},0})}{\partial z_{\mathbf{k},0}} - \sum_{\mathbf{k}\in\mathbf{G}\setminus\mathbf{0}} \frac{\partial \tilde{w}}{\partial \pi_\mathbf{k}}\frac{1}{N}\frac{\partial \pi(z_\bullet, \mathbf{z}_{\mathbf{0},0}, \mathbf{n}_{\mathbf{0},0})}{\partial z_{\mathbf{k},0}},
\end{aligned} \tag{A-38}
$$

where the first equality follows from taking the derivative to both sides of eq. (A-23); the second equality follows from applying the chain rule; the third equality follows from substituting eqs. (A-28), (A-31) and (A-32); and the last equality follows from distributing and rearranging terms.

Third, the derivative with respect to the average phenotype in any patch $\mathbf{j} \neq \mathbf{0}$ is

$$\begin{aligned}
\frac{\partial w(z_\bullet, \mathbf{z_0}, \mathbf{n_{0,0}})}{\partial z_{\mathbf{j},0}} &= \frac{\partial \tilde{w}(\boldsymbol{\pi}(z_\bullet, \mathbf{z_{0,0}}, \mathbf{n_{0,0}}))}{\partial z_{\mathbf{j},0}} \\
&= \frac{\partial \tilde{w}}{\partial \pi_\bullet}\frac{\partial \pi_\bullet}{\partial z_{\mathbf{j},0}} + \frac{\partial \tilde{w}}{\partial \pi_\mathbf{j}}\frac{\partial \pi_\mathbf{j}}{\partial z_{\mathbf{j},0}} + \sum_{\mathbf{k}\in\mathbf{G}\setminus\mathbf{j}} \frac{\partial \tilde{w}}{\partial \pi_\mathbf{k}}\frac{\partial \pi_\mathbf{k}}{\partial z_{\mathbf{j},0}} \\
&= \frac{\partial \tilde{w}}{\partial \pi_\bullet}\frac{\partial \pi(z_\bullet, \mathbf{z_{0,0}}, \mathbf{n_{0,0}})}{\partial z_{\mathbf{j},0}} + \frac{\partial \tilde{w}}{\partial \pi_\mathbf{j}}\left(\frac{\partial \pi(z_\bullet, \mathbf{z_{0,0}}, \mathbf{n_{0,0}})}{\partial z_\bullet} + \frac{\partial \pi(z_\bullet, \mathbf{z_{0,0}}, \mathbf{n_{0,0}})}{\partial z_{\mathbf{0},0}}\right) \\
&+ \sum_{\mathbf{k}\in\mathbf{G}\setminus\mathbf{j}} \frac{\partial \tilde{w}}{\partial \pi_\mathbf{k}}\frac{\partial \pi(z_\bullet, \mathbf{z_{0,0}}, \mathbf{n_{0,0}})}{\partial z_{\mathbf{j-k},0}} \\
&= \frac{\partial \tilde{w}}{\partial \pi_\bullet}\frac{\partial \pi(z_\bullet, \mathbf{z_{0,0}}, \mathbf{n_{0,0}})}{\partial z_{\mathbf{j},0}} + \frac{\partial \tilde{w}}{\partial \pi_\mathbf{j}}\frac{\partial \pi(z_\bullet, \mathbf{z_{0,0}}, \mathbf{n_{0,0}})}{\partial z_\bullet} + \sum_{\mathbf{k}\in\mathbf{G}} \frac{\partial \tilde{w}}{\partial \pi_\mathbf{k}}\frac{\partial \pi(z_\bullet, \mathbf{z_{0,0}}, \mathbf{n_{0,0}})}{\partial z_{\mathbf{j-k},0}},
\end{aligned} \quad \text{(A-39)}$$

where the second equality follows from applying the chain rule; the third equality follows from using the definition of $\pi_\bullet$ (eq. A-24) and substituting eq. (A-33) and eq. (A-34); and the fourth and last equality follows from rearranging.

Finally, the derivative with respect to the state variable in patch $\mathbf{j}$ is

$$\begin{aligned}
\frac{\partial w(z_\bullet, \mathbf{z_{0,0}}, \mathbf{n_{0,0}})}{\partial n_{\mathbf{j},0}} &= \frac{\partial \tilde{w}(\boldsymbol{\pi}(z_\bullet, \mathbf{z_{0,0}}, \mathbf{n_{0,0}}))}{\partial n_{\mathbf{j},0}} \\
&= \frac{\partial \tilde{w}}{\partial \pi_\bullet}\frac{\partial \pi_\bullet}{\partial n_{\mathbf{j},0}} + \sum_{\mathbf{k}\in\mathbf{G}} \frac{\partial \tilde{w}}{\partial \pi_\mathbf{k}}\frac{\partial \pi_\mathbf{k}}{\partial n_{\mathbf{j},0}} \\
&= \frac{\partial \tilde{w}}{\partial \pi_\bullet}\frac{\partial \pi(z_\bullet, \mathbf{z_{0,0}}, \mathbf{n_{0,0}})}{\partial n_{\mathbf{j},0}} + \sum_{\mathbf{k}\in\mathbf{G}} \frac{\partial \tilde{w}}{\partial \pi_\mathbf{k}}\frac{\partial \pi(z_\bullet, \mathbf{z_{0,0}}, \mathbf{n_{0,0}})}{\partial n_{\mathbf{j-k},0}},
\end{aligned} \quad \text{(A-40)}$$

where the second equality follows from applying the chain rule; and the third equality follows from substituting eqs. (A-35)–(A-36).

Let us denote by

$$\lambda_\mathbf{j} = -\frac{\partial \tilde{w}}{\partial \pi_\mathbf{j}} \Big/ \frac{\partial \tilde{w}}{\partial \pi_\bullet} \quad \text{(A-41)}$$

the coefficient of fitness interdependence between individuals in the focal patch and individuals in patch $\mathbf{j}$. We can express $s_\mathrm{W}(z)$ in terms of these coefficients of fitness interdependence and in terms of the derivatives of the fitness function with respect to the phenotypes of different actors in the following way. Substituting eqs. (A-37)–(A-39) into eq. (6a), factoring $\partial\tilde{w}/\partial\pi_\bullet$, and making use of (A-41), we

obtain

$$
\begin{aligned}
s_{\mathrm{w}}(z) &= \frac{\partial w}{\partial z_{\bullet}} + \frac{\partial w}{\partial z_{\mathbf{0},0}} R_{\mathbf{0},0} + \sum_{\mathbf{j}\in\mathbb{G}\setminus\mathbf{0}} \frac{\partial w}{\partial z_{\mathbf{j},0}} R_{\mathbf{j},0} \\
&= \underbrace{\frac{\partial \tilde{w}}{\partial \pi_{\bullet}} \frac{\partial \pi}{\partial z_{\bullet}} + \frac{\partial \tilde{w}}{\partial \pi_{\mathbf{0}}} \frac{1}{N-1} \frac{\partial \pi}{\partial z_{\mathbf{0},0}} + \sum_{\mathbf{k}\in\mathbb{G}\setminus\mathbf{0}} \frac{\partial \tilde{w}}{\partial \pi_{\mathbf{k}}} \frac{1}{N} \frac{\partial \pi}{\partial z_{\mathbf{k},0}}}_{\frac{\partial w}{\partial z_{\bullet}}} \\
&+ \underbrace{\left[\frac{\partial \tilde{w}}{\partial \pi_{\bullet}} \frac{\partial \pi}{\partial z_{\mathbf{0},0}} + \frac{\partial \tilde{w}}{\partial \pi_{0}} \left(\frac{\partial \pi}{\partial z_{\bullet}} - \frac{1}{N-1} \frac{\partial \pi}{\partial z_{\mathbf{0},0}} \right) + \sum_{\mathbf{k}\in\mathbb{G}} \frac{\partial \tilde{w}}{\partial \pi_{\mathbf{k}}} \frac{\partial \pi}{\partial z_{\mathbf{k},0}} - \sum_{\mathbf{k}\in\mathbb{G}\setminus\mathbf{0}} \frac{\partial \tilde{w}}{\partial \pi_{\mathbf{k}}} \frac{1}{N} \frac{\partial \pi}{\partial z_{\mathbf{k},0}} \right] R_{\mathbf{0},0}}_{\frac{\partial w}{\partial z_{\mathbf{0},0}}} \\
&+ \sum_{\mathbf{j}\in\mathbb{G}\setminus\mathbf{0}} \underbrace{\left[\frac{\partial \tilde{w}}{\partial \pi_{\bullet}} \frac{\partial \pi}{\partial z_{\mathbf{j},0}} + \frac{\partial \tilde{w}}{\partial \pi_{\mathbf{j}}} \frac{\partial \pi}{\partial z_{\bullet}} + \sum_{\mathbf{k}\in\mathbb{G}} \frac{\partial \tilde{w}}{\partial \pi_{\mathbf{k}}} \frac{\partial \pi}{\partial z_{\mathbf{j}-\mathbf{k},0}} \right]}_{\frac{\partial w}{\partial z_{\mathbf{j},0}}} R_{\mathbf{j},0} \\
&= \frac{\partial \tilde{w}}{\partial \pi_{\bullet}} \Bigg\{ \frac{\partial \pi}{\partial z_{\bullet}} - \lambda_{\mathbf{0}} \frac{1}{N-1} \frac{\partial \pi}{\partial z_{\mathbf{0},0}} - \sum_{\mathbf{k}\in\mathbb{G}\setminus\mathbf{0}} \lambda_{\mathbf{k}} \frac{1}{N} \frac{\partial \pi}{\partial z_{\mathbf{k},0}} \\
&+ \left[\frac{\partial \pi}{\partial z_{\mathbf{0},0}} - \lambda_{\mathbf{0}} \left(\frac{\partial \pi}{\partial z_{\bullet}} - \frac{1}{N-1} \frac{\partial \pi}{\partial z_{\mathbf{0},0}} \right) + \sum_{\mathbf{k}\in\mathbb{G}\setminus\mathbf{0}} \lambda_{\mathbf{k}} \frac{1}{N} \frac{\partial \pi}{\partial z_{\mathbf{k},0}} - \sum_{\mathbf{k}\in\mathbb{G}} \lambda_{\mathbf{k}} \frac{\partial \pi}{\partial z_{\mathbf{k},0}} \right] R_{\mathbf{0},0} \\
&+ \sum_{\mathbf{j}\in\mathbb{G}\setminus\mathbf{0}} \left[\frac{\partial \pi}{\partial z_{\mathbf{j},0}} - \lambda_{\mathbf{j}} \frac{\partial \pi}{\partial z_{\bullet}} - \sum_{\mathbf{k}\in\mathbb{G}} \lambda_{\mathbf{k}} \frac{\partial \pi}{\partial z_{\mathbf{j}-\mathbf{k},0}} \right] R_{\mathbf{j},0} \Bigg\} \\
&= \frac{\partial \tilde{w}}{\partial \pi_{\bullet}} \Bigg\{ \frac{\partial \pi}{\partial z_{\bullet}} - \lambda_{\mathbf{0}} \frac{1}{N-1} \frac{\partial \pi}{\partial z_{\mathbf{0},0}} - \sum_{\mathbf{k}\in\mathbb{G}\setminus\mathbf{0}} \lambda_{\mathbf{k}} \frac{1}{N} \frac{\partial \pi}{\partial z_{\mathbf{k},0}} \\
&+ \left[\frac{\partial \pi}{\partial z_{\mathbf{0},0}} - \lambda_{\mathbf{0}} \frac{\partial \pi}{\partial z_{\bullet}} - \lambda_{\mathbf{0}} \frac{1}{N-1} \frac{\partial \pi}{\partial z_{\mathbf{0},0}} + \sum_{\mathbf{k}\in\mathbb{G}\setminus\mathbf{0}} \lambda_{\mathbf{k}} \frac{1}{N} \frac{\partial \pi}{\partial z_{\mathbf{k},0}} - \lambda_{\mathbf{0}} \frac{\partial \pi}{\partial z_{\mathbf{0},0}} - \sum_{\mathbf{k}\in\mathbb{G}\setminus\mathbf{0}} \lambda_{\mathbf{k}} \frac{\partial \pi}{\partial z_{\mathbf{k},0}} \right] R_{\mathbf{0},0} \\
&+ \sum_{\mathbf{j}\in\mathbb{G}\setminus\mathbf{0}} \left[\frac{\partial \pi}{\partial z_{\mathbf{j},0}} - \lambda_{\mathbf{j}} \frac{\partial \pi}{\partial z_{\bullet}} - \lambda_{\mathbf{j}} \frac{\partial \pi}{\partial z_{\mathbf{0},0}} - \sum_{\mathbf{k}\in\mathbb{G}\setminus\mathbf{j}} \lambda_{\mathbf{k}} \frac{\partial \pi}{\partial z_{\mathbf{j}-\mathbf{k},0}} \right] R_{\mathbf{j},0} \Bigg\}. \qquad \text{(A-42)}
\end{aligned}
$$

Collecting terms and simplifying, we further get

$$
\begin{aligned}
s_{\mathrm{w}}(z) = \frac{\partial \tilde{w}}{\partial \pi_{\bullet}} \Bigg\{ & \frac{\partial \pi}{\partial z_{\bullet}} \left(1 - \sum_{\mathbf{j}\in\mathbb{G}} \lambda_{\mathbf{j}} R_{\mathbf{j},0} \right) + \frac{\partial \pi}{\partial z_{\mathbf{0},0}} \left[R_{\mathbf{0},0} - \lambda_{\mathbf{0}} \frac{1}{N-1} (1 - R_{\mathbf{0},0}) - \sum_{\mathbf{j}\in\mathbb{G}} \lambda_{\mathbf{j}} R_{\mathbf{j},0} \right] \\
& + \sum_{\mathbf{k}\in\mathbb{G}\setminus\mathbf{0}} \frac{\partial \pi}{\partial z_{\mathbf{k},0}} \left[R_{\mathbf{k},0} - \frac{1}{N} \lambda_{\mathbf{k}} (1 - R_{\mathbf{0},0}) \right] - \underbrace{\sum_{\mathbf{j}\in\mathbb{G}} \sum_{\mathbf{k}\in\mathbb{G}\setminus\mathbf{j}} \lambda_{\mathbf{k}} \frac{\partial \pi}{\partial z_{\mathbf{j}-\mathbf{k},0}} R_{\mathbf{j},0}}_{U} \Bigg\}. \qquad \text{(A-43)}
\end{aligned}
$$

To further simplify this expression, note that the underbraced term can be rewritten as

$$
\begin{aligned}
U &= \sum_{\mathbf{j}\in\mathbb{G}} \sum_{\mathbf{k}\in\mathbb{G}\setminus\mathbf{j}} \lambda_{\mathbf{k}} \frac{\partial \pi}{\partial z_{\mathbf{j}-\mathbf{k},0}} R_{\mathbf{j},0} \\
&= \sum_{\mathbf{j}\in\mathbb{G}} \sum_{\mathbf{k}\in\mathbb{G}} \lambda_{\mathbf{k}} \frac{\partial \pi}{\partial z_{\mathbf{j}-\mathbf{k},0}} R_{\mathbf{j},0} - \sum_{\mathbf{j}\in\mathbb{G}} \lambda_{\mathbf{j}} \frac{\partial \pi}{\partial z_{\mathbf{0},0}} R_{\mathbf{j},0} \\
&= \sum_{\mathbf{k}\in\mathbb{G}} \lambda_{\mathbf{k}} \sum_{\mathbf{j}\in\mathbb{G}} \frac{\partial \pi}{\partial z_{\mathbf{j}-\mathbf{k},0}} R_{\mathbf{j},0} - \frac{\partial \pi}{\partial z_{\mathbf{0},0}} \sum_{\mathbf{j}\in\mathbb{G}} \lambda_{\mathbf{j}} R_{\mathbf{j},0} \\
&= \sum_{\mathbf{k}\in\mathbb{G}} \lambda_{\mathbf{k}} \sum_{\mathbf{j}\in\mathbb{G}} \frac{\partial \pi}{\partial z_{\mathbf{j},0}} R_{\mathbf{j}-\mathbf{k},0} - \frac{\partial \pi}{\partial z_{\mathbf{0},0}} \sum_{\mathbf{j}\in\mathbb{G}} \lambda_{\mathbf{j}} R_{\mathbf{j},0} \\
&= \sum_{\mathbf{j}\in\mathbb{G}} \frac{\partial \pi}{\partial z_{\mathbf{j},0}} \sum_{\mathbf{k}\in\mathbb{G}} \lambda_{\mathbf{k}} R_{\mathbf{j}-\mathbf{k},0} - \frac{\partial \pi}{\partial z_{\mathbf{0},0}} \sum_{\mathbf{j}\in\mathbb{G}} \lambda_{\mathbf{j}} R_{\mathbf{j},0} \\
&= \sum_{\mathbf{j}\in\mathbb{G}\setminus\mathbf{0}} \frac{\partial \pi}{\partial z_{\mathbf{j},0}} \sum_{\mathbf{k}\in\mathbb{G}} \lambda_{\mathbf{k}} R_{\mathbf{j}-\mathbf{k},0} \\
&= \sum_{\mathbf{k}\in\mathbb{G}\setminus\mathbf{0}} \frac{\partial \pi}{\partial z_{\mathbf{k},0}} \sum_{\mathbf{j}\in\mathbb{G}} \lambda_{\mathbf{j}} R_{\mathbf{j}-\mathbf{k},0},
\end{aligned}
\tag{A-44}
$$

where the third line follows from the identity

$$
\sum_{\mathbf{j}\in\mathbb{G}} f_{\mathbf{j}} g_{\mathbf{k}-\mathbf{j}} = \sum_{\mathbf{j}\in\mathbb{G}} f_{\mathbf{k}-\mathbf{j}} g_{\mathbf{j}},
\tag{A-45}
$$

and the last line follows from changing the dummy variables and from the symmetry of the relatedness coefficients (i.e. the fact that $R_{-\mathbf{k},0} = R_{\mathbf{k},0}$ holds for all $\mathbf{k} \in \mathbb{G}$).

Substituting (A-44) into (A-43) and simplifying we obtain

$$
\begin{aligned}
s_{\mathrm{w}}(z) &= \frac{\partial \tilde{w}}{\partial \pi_\bullet} \left\{ \frac{\partial \pi}{\partial z_\bullet} \left(1 - \sum_{\mathbf{j}\in\mathbb{G}} \lambda_{\mathbf{j}} R_{\mathbf{j},0} \right) + \frac{\partial \pi}{\partial z_{\mathbf{0},0}} \left[R_{\mathbf{0},0} - \lambda_{\mathbf{0}} \frac{1}{N-1} (1 - R_{\mathbf{0},0}) - \sum_{\mathbf{j}\in\mathbb{G}} \lambda_{\mathbf{j}} R_{\mathbf{j},0} \right] \right. \\
&\quad \left. + \sum_{\mathbf{k}\in\mathbb{G}\setminus\mathbf{0}} \frac{\partial \pi}{\partial z_{\mathbf{k},0}} \left[R_{\mathbf{k},0} - \frac{1}{N} \lambda_{\mathbf{k}} (1 - R_{\mathbf{0},0}) - \sum_{\mathbf{j}\in\mathbb{G}} \lambda_{\mathbf{j}} R_{\mathbf{j}-\mathbf{k},0} \right] \right\} \\
&= \frac{\partial \tilde{w}}{\partial \pi_\bullet} \left(1 - \sum_{\mathbf{j}\in\mathbb{G}} \lambda_{\mathbf{j}} R_{\mathbf{j},0} \right) \left\{ \frac{\partial \pi}{\partial z_\bullet} + \frac{R_{\mathbf{0},0} - \lambda_{\mathbf{0}} \frac{1}{N-1} (1 - R_{\mathbf{0},0}) - \sum_{\mathbf{j}\in\mathbb{G}} \lambda_{\mathbf{j}} R_{\mathbf{j},0}}{1 - \sum_{\mathbf{j}\in\mathbb{G}} \lambda_{\mathbf{j}} R_{\mathbf{j},0}} \frac{\partial \pi}{\partial z_{\mathbf{0},0}} \right. \\
&\quad \left. + \sum_{\mathbf{k}\in\mathbb{G}\setminus\mathbf{0}} \frac{R_{\mathbf{k},0} - \frac{1}{N} \lambda_{\mathbf{k}} (1 - R_{\mathbf{0},0}) - \sum_{\mathbf{j}\in\mathbb{G}} \lambda_{\mathbf{j}} R_{\mathbf{j}-\mathbf{k},0}}{1 - \sum_{\mathbf{j}\in\mathbb{G}} \lambda_{\mathbf{j}} R_{\mathbf{j},0}} \frac{\partial \pi}{\partial z_{\mathbf{k},0}} \right\} \\
&= L \left\{ \frac{\partial \pi}{\partial z_\bullet} + \sum_{\mathbf{k}\in\mathbb{G}} \kappa_{\mathbf{k},0} \frac{\partial \pi}{\partial z_{\mathbf{k},0}} \right\},
\end{aligned}
\tag{A-46}
$$

where the second equality follows from factoring $\frac{\partial \tilde{w}}{\partial \pi_\bullet} \left(1 - \sum_{\mathbf{j}\in\mathbb{G}} \lambda_{\mathbf{j}} R_{\mathbf{j},0} \right)$, and the final equality follows from defining

$$
L = \frac{\partial \tilde{w}}{\partial \pi_\bullet} \left(1 - \sum_{\mathbf{j}\in\mathbb{G}} \lambda_{\mathbf{j}} R_{\mathbf{j},0} \right),
\tag{A-47}
$$

$$\kappa_{\mathbf{0},0} = \frac{R_{\mathbf{0},0} - \frac{1}{N-1}\lambda_{\mathbf{0}}(1-R_{\mathbf{0},0}) - \sum_{\mathbf{j}\in G}\lambda_{\mathbf{j}}R_{\mathbf{j},0}}{1-\sum_{\mathbf{j}\in G}\lambda_{\mathbf{j}}R_{\mathbf{j},0}}, \tag{A-48}$$

and

$$\kappa_{\mathbf{k},0} = \frac{R_{\mathbf{k},0} - \frac{1}{N}\lambda_{\mathbf{k}}(1-R_{\mathbf{0},0}) - \sum_{\mathbf{j}\in G}\lambda_{\mathbf{j}}R_{\mathbf{j}-\mathbf{k},0}}{1-\sum_{\mathbf{j}\in G}\lambda_{\mathbf{j}}R_{\mathbf{j},0}} \tag{A-49}$$

for $\mathbf{k} \neq \mathbf{0}$. Eq. (A-46) corresponds to the expression for $s_{\mathrm{w}}(z)$ in eq. (18) of the main text, as required, by rearranging the numerators of eq. (A-48) and eq. (A-49). The representation of the numerators in eqs. (A-48)–(A-49) are useful for computations, while those in eq. (19) are more amenable to interpretation.

Note that in the infinite island model of dispersal, $R_{\mathbf{j},0} = 0$ for all $\mathbf{j} \neq \mathbf{0}$. In this case, $\kappa_{\mathbf{0},0}$ (eq. A-48) reduces to eq. 22 of [7] as it should. This provides a consistency check of our derivation.

Let us turn to express $s_{\mathrm{e}}(z)$ in terms of the coefficients of fitness interdependence. From eq. (11) and after substituting (A-40), factoring $\partial\tilde{w}/\partial\pi_{\bullet}$, and making use of eq. (A-41), we obtain

$$\begin{aligned}
s_{\mathrm{e}}(z) &= N\sum_{t=1}^{\infty}\sum_{\mathbf{k}\in G}\sum_{\mathbf{j}\in G} e_{\mathbf{j}-\mathbf{k},t}R_{\mathbf{k},t}\frac{\partial w}{\partial n_{\mathbf{j},0}} \\
&= N\sum_{t=1}^{\infty}\sum_{\mathbf{k}\in G}\sum_{\mathbf{j}\in G} e_{\mathbf{j}-\mathbf{k},t}R_{\mathbf{k},t}\left[\frac{\partial\tilde{w}}{\partial\pi_{\bullet}}\frac{\partial\pi}{\partial n_{\mathbf{j},0}} + \sum_{\mathbf{i}\in G}\frac{\partial\tilde{w}}{\partial\pi_{\mathbf{i}}}\frac{\partial\pi}{\partial n_{\mathbf{j}-\mathbf{i},0}}\right] \\
&= N\frac{\partial\tilde{w}}{\partial\pi_{\bullet}}\sum_{t=1}^{\infty}\sum_{\mathbf{k}\in G}\sum_{\mathbf{j}\in G} e_{\mathbf{j}-\mathbf{k},t}R_{\mathbf{k},t}\left[\frac{\partial\pi}{\partial n_{\mathbf{j},0}} - \sum_{\mathbf{i}\in G}\lambda_{\mathbf{i}}\frac{\partial\pi}{\partial n_{\mathbf{j}-\mathbf{i},0}}\right].
\end{aligned} \tag{A-50}$$

By rearranging terms and applying the identity (A-45), we can rewrite this expression as

$$\begin{aligned}
s_{\mathrm{e}}(z) &= N\frac{\partial\tilde{w}}{\partial\pi_{\bullet}}\sum_{t=1}^{\infty}\left[\sum_{\mathbf{j}\in G}\frac{\partial\pi}{\partial n_{\mathbf{j},0}}\sum_{\mathbf{k}\in G}e_{\mathbf{j}-\mathbf{k},t}R_{\mathbf{k},t} - \sum_{\mathbf{j}\in G}\sum_{\mathbf{i}\in G}\lambda_{\mathbf{i}}\frac{\partial\pi}{\partial n_{\mathbf{j}-\mathbf{i},0}}\sum_{\mathbf{k}\in G}e_{\mathbf{j}-\mathbf{k},t}R_{\mathbf{k},t}\right] \\
&= N\frac{\partial\tilde{w}}{\partial\pi_{\bullet}}\sum_{t=1}^{\infty}\left[\sum_{\mathbf{j}\in G}\frac{\partial\pi}{\partial n_{\mathbf{j},0}}\sum_{\mathbf{k}\in G}e_{\mathbf{k},t}R_{\mathbf{j}-\mathbf{k},t} - \sum_{\mathbf{j}\in G}\sum_{\mathbf{i}\in G}\lambda_{\mathbf{i}}\frac{\partial\pi}{\partial n_{\mathbf{j}-\mathbf{i},0}}\sum_{\mathbf{k}\in G}e_{\mathbf{k},t}R_{\mathbf{j}-\mathbf{k},t}\right] \\
&= N\frac{\partial\tilde{w}}{\partial\pi_{\bullet}}\sum_{t=1}^{\infty}\left[\sum_{\mathbf{i}\in G}\frac{\partial\pi}{\partial n_{\mathbf{i},0}}\sum_{\mathbf{k}\in G}e_{\mathbf{k},t}R_{\mathbf{i}-\mathbf{k},t} - \sum_{\mathbf{j}\in G}\sum_{\mathbf{k}\in G}e_{\mathbf{k},t}R_{\mathbf{j}-\mathbf{k},t}\sum_{\mathbf{i}\in G}\lambda_{\mathbf{i}}\frac{\partial\pi}{\partial n_{\mathbf{j}-\mathbf{i},0}}\right] \\
&= N\frac{\partial\tilde{w}}{\partial\pi_{\bullet}}\sum_{t=1}^{\infty}\left[\sum_{\mathbf{i}\in G}\frac{\partial\pi}{\partial n_{\mathbf{i},0}}\sum_{\mathbf{k}\in G}e_{\mathbf{k},t}R_{\mathbf{i}-\mathbf{k},t} - \sum_{\mathbf{j}\in G}\sum_{\mathbf{k}\in G}e_{\mathbf{k},t}R_{\mathbf{j}-\mathbf{k},t}\sum_{\mathbf{i}\in G}\lambda_{\mathbf{j}-\mathbf{i}}\frac{\partial\pi}{\partial n_{\mathbf{i},0}}\right] \\
&= N\frac{\partial\tilde{w}}{\partial\pi_{\bullet}}\sum_{t=1}^{\infty}\left[\sum_{\mathbf{i}\in G}\frac{\partial\pi}{\partial n_{\mathbf{i},0}}\sum_{\mathbf{k}\in G}e_{\mathbf{k},t}R_{\mathbf{i}-\mathbf{k},t} - \sum_{\mathbf{i}\in G}\frac{\partial\pi}{\partial n_{\mathbf{i},0}}\sum_{\mathbf{k}\in G}e_{\mathbf{k},t}\sum_{\mathbf{j}\in G}R_{\mathbf{j}-\mathbf{k},t}\lambda_{\mathbf{j}-\mathbf{i}}\right] \\
&= N\frac{\partial\tilde{w}}{\partial\pi_{\bullet}}\sum_{t=1}^{\infty}\sum_{\mathbf{i}\in G}\frac{\partial\pi}{\partial n_{\mathbf{i},0}}\sum_{\mathbf{k}\in G}e_{\mathbf{k},t}\left[R_{\mathbf{i}-\mathbf{k},t} - \sum_{\mathbf{j}\in G}\lambda_{\mathbf{j}-\mathbf{i}}R_{\mathbf{j}-\mathbf{k},t}\right].
\end{aligned} \tag{A-51}$$

Using the symmetry of the relatedness coefficients and changing the summation indices we further

get

$$
\begin{aligned}
s_{\mathrm{e}}(z) &= N\frac{\partial\tilde{w}}{\partial\pi_{\bullet}}\sum_{t=1}^{\infty}\sum_{\mathbf{i}\in\mathbb{G}}\frac{\partial\pi}{\partial n_{\mathbf{i},0}}\sum_{\mathbf{k}\in\mathbb{G}}e_{\mathbf{k},t}\left[R_{\mathbf{k}-\mathbf{i},t}-\sum_{\mathbf{j}\in\mathbb{G}}\lambda_{\mathbf{j}}R_{\mathbf{j}-\mathbf{k}+\mathbf{i},t}\right] \\
&= N\frac{\partial\tilde{w}}{\partial\pi_{\bullet}}\sum_{t=1}^{\infty}\sum_{\mathbf{i}\in\mathbb{G}}\frac{\partial\pi}{\partial n_{\mathbf{i},0}}\sum_{\mathbf{k}\in\mathbb{G}}e_{\mathbf{k}-\mathbf{i},t}\left[R_{\mathbf{k},t}-\sum_{\mathbf{j}\in\mathbb{G}}\lambda_{\mathbf{j}}R_{\mathbf{j}-\mathbf{k},t}\right] \\
&= N\frac{\partial\tilde{w}}{\partial\pi_{\bullet}}\left(1-\sum_{\mathbf{j}\in\mathbb{G}}\lambda_{\mathbf{j}}R_{\mathbf{j},0}\right)\sum_{t=1}^{\infty}\sum_{\mathbf{k}\in\mathbb{G}}\sum_{\mathbf{i}\in\mathbb{G}}\frac{\partial\pi}{\partial n_{\mathbf{i},0}}e_{\mathbf{k}-\mathbf{i},t}\frac{R_{\mathbf{k},t}-\sum_{\mathbf{j}\in\mathbb{G}}\lambda_{\mathbf{j}}R_{\mathbf{j}-\mathbf{k},t}}{1-\sum_{\mathbf{j}\in\mathbb{G}}\lambda_{\mathbf{j}}R_{\mathbf{j},0}} \\
&= LN\sum_{t=1}^{\infty}\sum_{\mathbf{k}\in\mathbb{G}}\sum_{\mathbf{i}\in\mathbb{G}}\frac{\partial\pi}{\partial n_{\mathbf{i},0}}e_{\mathbf{k}-\mathbf{i},t}\kappa_{\mathbf{k},t},
\end{aligned}
\tag{A-52}
$$

where the second-to-last line follows from multiplying and dividing by $1-\sum_{\mathbf{j}\in\mathbb{G}}\lambda_{\mathbf{j}}R_{\mathbf{j},0}$, and the last line follows from identifying L (A-47) and defining

$$
\kappa_{\mathbf{k},t}=\frac{R_{\mathbf{k},t}-\sum_{\mathbf{j}\in\mathbb{G}}\lambda_{\mathbf{j}}R_{\mathbf{j}-\mathbf{k},t}}{1-\sum_{\mathbf{j}\in\mathbb{G}}\lambda_{\mathbf{j}}R_{\mathbf{j},0}}\quad\text{for } t>0. \tag{A-53}
$$

Eq. (A-52) corresponds to the expression for $s_{\mathrm{e}}(z)$ in eq. (18) of the main text, as required.

Adding the intra- (eq. A-46) and inter-temporal (eq. A-52) components of the selection gradient, we obtain

$$
\begin{aligned}
s(z) &= s_{\mathrm{w}}(z)+s_{\mathrm{e}}(z) \\
&= L\left\{\frac{\partial\pi}{\partial z_{\bullet}}+\sum_{\mathbf{k}\in\mathbb{G}}\kappa_{\mathbf{k},0}\frac{\partial\pi}{\partial z_{\mathbf{k},0}}+N\sum_{t=1}^{\infty}\sum_{\mathbf{k}\in\mathbb{G}}\sum_{\mathbf{i}\in\mathbb{G}}\frac{\partial\pi}{\partial n_{\mathbf{i},0}}e_{\mathbf{k}-\mathbf{i},t}\kappa_{\mathbf{k},t}\right\},
\end{aligned}
\tag{A-54}
$$

overall.

For derivations to come, it is convenient to have the scaled-relatedness coefficients written in terms of the partial derivatives of $\tilde{w}$ with respect to the payoffs of different individuals. From eqs. (A-48), (A-49) and (A-53) and the definition of the coefficients of fitness interdependence (A-41) we have after rearrangements and multiplying numerators and denominators by $\partial\tilde{w}/\partial\pi_{\bullet}$:

$$
\kappa_{\mathbf{0},0}=\frac{R_{\mathbf{0},0}\frac{\partial\tilde{w}}{\partial\pi_{\bullet}}+\frac{\partial\tilde{w}}{\partial\pi_{\mathbf{0}}}\left[\frac{1}{N-1}(1-R_{\mathbf{0},0})+R_{\mathbf{0},0}\right]+\sum_{\mathbf{j}\in\mathbb{G}\backslash\mathbf{0}}\frac{\partial\tilde{w}}{\partial\pi_{\mathbf{j}}}R_{\mathbf{j},0}}{\frac{\partial\tilde{w}}{\partial\pi_{\bullet}}+\frac{\partial\tilde{w}}{\partial\pi_{\mathbf{0}}}R_{\mathbf{0},0}+\sum_{\mathbf{j}\in\mathbb{G}\backslash\mathbf{0}}\frac{\partial\tilde{w}}{\partial\pi_{\mathbf{j}}}R_{\mathbf{j},0}}, \tag{A-55}
$$

$$
\kappa_{\mathbf{k},0}=\frac{R_{\mathbf{k},t}\frac{\partial\tilde{w}}{\partial\pi_{\bullet}}+\frac{\partial\tilde{w}}{\partial\pi_{\mathbf{k}}}\frac{1}{N}(1-R_{\mathbf{0},0})+\sum_{\mathbf{j}\in\mathbb{G}}\frac{\partial\tilde{w}}{\partial\pi_{\mathbf{j}}}R_{\mathbf{j}-\mathbf{k},0}}{\frac{\partial\tilde{w}}{\partial\pi_{\bullet}}+\frac{\partial\tilde{w}}{\partial\pi_{\mathbf{0}}}R_{\mathbf{0},0}+\sum_{\mathbf{j}\in\mathbb{G}\backslash\mathbf{0}}\frac{\partial\tilde{w}}{\partial\pi_{\mathbf{j}}}R_{\mathbf{j},0}}\quad\text{for } \mathbf{k}\neq\mathbf{0}, \tag{A-56}
$$

and

$$
\kappa_{\mathbf{k},t}=\frac{R_{\mathbf{k},t}\frac{\partial\tilde{w}}{\partial\pi_{\bullet}}+\sum_{\mathbf{j}\in\mathbb{G}}\frac{\partial\tilde{w}}{\partial\pi_{\mathbf{j}}}R_{\mathbf{j}-\mathbf{k},t}}{\frac{\partial\tilde{w}}{\partial\pi_{\bullet}}+\frac{\partial\tilde{w}}{\partial\pi_{\mathbf{0}}}R_{\mathbf{0},0}+\sum_{\mathbf{j}\in\mathbb{G}\backslash\mathbf{0}}\frac{\partial\tilde{w}}{\partial\pi_{\mathbf{j}}}R_{\mathbf{j},0}}\quad\text{for } t>0. \tag{A-57}
$$

Appendix E Explicit expressions for scaled relatedness

Here, we derive the explicit expression for scaled relatedness $\kappa_{\mathbf{k},t}$ given by eq. (III.A) in Box 3, which is based on the assumption that individual fitness can be written as eq. (17); that is

$$\tilde{w}(\boldsymbol{\pi}) = \mathsf{s}\left(\pi_{\bullet}\right) + \sum_{\mathbf{i}\in\mathbb{G}} m_{\mathbf{i}} \left[1 - \mathsf{s}^{\mathrm{R}}\left(\pi_{\mathbf{i}}\right)\right] \frac{\mathsf{f}(\pi_{\bullet})}{\sum_{\mathbf{j}\in\mathbb{G}} m_{\mathbf{i}-\mathbf{j}} \mathsf{f}^{\mathrm{R}}(\pi_{\mathbf{j}})}, \tag{A-58}$$

where

$$\mathsf{s}^{\mathrm{R}}(\pi_{\mathbf{i}}) = \begin{cases} \frac{1}{N}\mathsf{s}(\pi_{\bullet}) + \frac{N-1}{N}\mathsf{s}(\pi_{\mathbf{0}}) & \text{if } \mathbf{i} = \mathbf{0} \\ \mathsf{s}(\pi_{\mathbf{i}}) & \text{otherwise} \end{cases} \tag{A-59}$$

and

$$\mathsf{f}^{\mathrm{R}}(\pi_{\mathbf{i}}) = \begin{cases} \frac{1}{N}\mathsf{f}(\pi_{\bullet}) + \frac{N-1}{N}\mathsf{f}(\pi_{\mathbf{0}}) & \text{if } \mathbf{i} = \mathbf{0} \\ \mathsf{f}(\pi_{\mathbf{i}}) & \text{otherwise.} \end{cases} \tag{A-60}$$

We proceed in three steps. First, we calculate payoff derivatives and the coefficients of fitness interdependence in terms of demographic parameters (Appendix E.1). Second, we calculate expressions for the scaled relatedness coefficients in terms of relatedness coefficients (Appendix E.2). Third, starting from these expressions, we calculate expressions for the scaled relatedness coefficients in terms of demographic parameters, obtaining eq. (III.A) shown in Box 3 (Appendix E.3). Finally, in Appendix E.4, we use these results to get an expression for L (A-47) in terms of demographic parameters, which can be useful to have the magnitude (not just the sign) of the selection gradient.

Appendix E.1 Payoff derivatives and coefficients of fitness interdependence

Using the quotient rule of derivatives, and evaluating expressions at the resident trait value, the derivative of $\tilde{w}$ (A-58) with respect to the payoff of the focal individual $\pi_{\bullet}$ can be written as

$$\begin{aligned}
\frac{\partial \tilde{w}}{\partial \pi_{\bullet}} &= \frac{\partial \mathsf{s}\left(\pi_{\bullet}\right)}{\partial \pi_{\bullet}} + \sum_{\mathbf{i}\in\mathbb{G}} m_{\mathbf{i}} \frac{\partial}{\partial \pi_{\bullet}} \left\{ \frac{\left[1 - \mathsf{s}^{\mathrm{R}}\left(\pi_{\mathbf{i}}\right)\right] \mathsf{f}(\pi_{\bullet})}{\sum_{\mathbf{j}\in\mathbb{G}} m_{\mathbf{i}-\mathbf{j}} \mathsf{f}^{\mathrm{R}}(\pi_{\mathbf{j}})} \right\} \\
&= \mathsf{s}' + \sum_{\mathbf{i}\in\mathbb{G}} m_{\mathbf{i}} \frac{\frac{\partial}{\partial \pi_{\bullet}} \left\{ \left[1 - \mathsf{s}^{\mathrm{R}}\left(\pi_{\mathbf{i}}\right)\right] \mathsf{f}(\pi_{\bullet}) \right\} \sum_{\mathbf{j}\in\mathbb{G}} m_{\mathbf{i}-\mathbf{j}} \mathsf{f}^{\mathrm{R}}(\pi_{\mathbf{j}}) - \left[1 - \mathsf{s}^{\mathrm{R}}\left(\pi_{\mathbf{i}}\right)\right] \mathsf{f}(\pi_{\bullet}) \sum_{\mathbf{j}\in\mathbb{G}} m_{\mathbf{i}-\mathbf{j}} \frac{\partial \mathsf{f}^{\mathrm{R}}(\pi_{\mathbf{j}})}{\partial \pi_{\bullet}}}{\left[\sum_{\mathbf{j}\in\mathbb{G}} m_{\mathbf{i}-\mathbf{j}} \mathsf{f}^{\mathrm{R}}(\pi_{\mathbf{j}})\right]^2} \\
&= \mathsf{s}' + \sum_{\mathbf{i}\in\mathbb{G}} m_{\mathbf{i}} \frac{\frac{\partial}{\partial \pi_{\bullet}} \left\{ \left[1 - \mathsf{s}^{\mathrm{R}}\left(\pi_{\mathbf{i}}\right)\right] \mathsf{f}(\pi_{\bullet}) \right\} \mathsf{f} - (1-\mathsf{s})\mathsf{f} \sum_{\mathbf{j}\in\mathbb{G}} m_{\mathbf{i}-\mathbf{j}} \frac{\partial \mathsf{f}^{\mathrm{R}}(\pi_{\mathbf{j}})}{\partial \pi_{\bullet}}}{\mathsf{f}^2} \\
&= \mathsf{s}' + \sum_{\mathbf{i}\in\mathbb{G}} m_{\mathbf{i}} \frac{\frac{\partial}{\partial \pi_{\bullet}} \left\{ \left[1 - \mathsf{s}^{\mathrm{R}}\left(\pi_{\mathbf{i}}\right)\right] \mathsf{f}(\pi_{\bullet}) \right\}}{\mathsf{f}} - \sum_{\mathbf{i}\in\mathbb{G}} m_{\mathbf{i}} \frac{(1-\mathsf{s}) \sum_{\mathbf{j}\in\mathbb{G}} m_{\mathbf{i}-\mathbf{j}} \frac{\partial \mathsf{f}^{\mathrm{R}}(\pi_{\mathbf{j}})}{\partial \pi_{\bullet}}}{\mathsf{f}},
\end{aligned}$$

where we have set $\mathsf{s}' = \partial \mathsf{s}\left(\pi_{\bullet}\right) / \partial \pi_{\bullet}$, and used the fact that $\sum_{\mathbf{j}\in\mathbb{G}} m_{\mathbf{i}-\mathbf{j}} = 1$ for all $\mathbf{i} \in \mathbb{G}$.

Substituting (A-59), noting that

$$\frac{\partial \mathsf{s}^{\mathrm{R}}(\pi_{\mathbf{i}})}{\partial \pi_{\bullet}} = \begin{cases} \frac{1}{N}\mathsf{s}' & \text{if } \mathbf{i} = \mathbf{0} \\ 0 & \text{otherwise,} \end{cases} \tag{A-61}$$

and

$$\frac{\partial \mathsf{f}^{\mathrm{R}}(\pi_{\mathbf{i}})}{\partial \pi_{\bullet}} = \begin{cases} \frac{1}{N}\mathsf{f}' & \text{if } \mathbf{i} = \mathbf{0} \\ 0 & \text{otherwise} \end{cases} \tag{A-62}$$

hold, and setting $\mathsf{f}' = \partial \mathsf{f}\left(\pi_{\bullet}\right)/\partial \pi_{\bullet}$, we further get

$$\begin{aligned}
\frac{\partial \tilde{w}}{\partial \pi_{\bullet}} &= \mathsf{s}' + m_{\mathbf{0}} \frac{\frac{\partial}{\partial \pi_{\bullet}}\left\{\left[1 - \frac{1}{N}\mathsf{s}(\pi_{\bullet}) - \frac{N-1}{N}\mathsf{s}(\pi_{\mathbf{0}})\right]\mathsf{f}(\pi_{\bullet})\right\}}{\mathsf{f}} + \sum_{\mathbf{i}\in\mathbf{G}\setminus\mathbf{0}} m_{\mathbf{i}} \frac{\frac{\partial}{\partial \pi_{\bullet}}\left\{\left[1 - \mathsf{s}\left(\pi_{\mathbf{i}}\right)\right]\mathsf{f}(\pi_{\bullet})\right\}}{\mathsf{f}} \\
&\quad - \sum_{\mathbf{i}\in\mathbf{G}} m_{\mathbf{i}} \frac{(1-\mathsf{s})\sum_{\mathbf{j}\in\mathbf{G}} m_{\mathbf{i}-\mathbf{j}} \frac{\partial \mathsf{f}^{\mathrm{R}}(\pi_{\mathbf{j}})}{\partial \pi_{\bullet}}}{\mathsf{f}} \\
&= \mathsf{s}' + m_{\mathbf{0}} \frac{(1-\mathsf{s})\mathsf{f}' - \frac{1}{N}\mathsf{s}'\mathsf{f}}{\mathsf{f}} + \sum_{\mathbf{i}\in\mathbf{G}\setminus\mathbf{0}} m_{\mathbf{i}} \frac{(1-\mathsf{s})\mathsf{f}'}{\mathsf{f}} - \sum_{\mathbf{i}\in\mathbf{G}} m_{\mathbf{i}} \frac{(1-\mathsf{s})m_{\mathbf{i}}\frac{1}{N}\mathsf{f}'}{\mathsf{f}} \\
&= \mathsf{s}' - m_{\mathbf{0}}\frac{1}{N}\mathsf{s}' + m_{\mathbf{0}}\frac{(1-\mathsf{s})\mathsf{f}'}{\mathsf{f}} + \sum_{\mathbf{i}\in\mathbf{G}\setminus\mathbf{0}} m_{\mathbf{i}} \frac{(1-\mathsf{s})\mathsf{f}'}{\mathsf{f}} - \frac{1}{N}(1-\mathsf{s})\frac{\mathsf{f}'}{\mathsf{f}}\sum_{\mathbf{i}\in\mathbf{G}} m_{\mathbf{i}}^2 \\
&= \mathsf{s}'\left(1 - m_{\mathbf{0}}\frac{1}{N}\right) + (1-\mathsf{s})\frac{\mathsf{f}'}{\mathsf{f}}\sum_{\mathbf{i}\in\mathbf{G}} m_{\mathbf{i}} - \frac{1}{N}(1-\mathsf{s})\frac{\mathsf{f}'}{\mathsf{f}}\sum_{\mathbf{i}\in\mathbf{G}} m_{\mathbf{i}}^2 \\
&= \left(1 - \frac{1}{N}m_{\mathbf{0}}\right)\mathsf{s}' + \left(1 - \frac{1}{N}\sum_{\mathbf{i}\in\mathbf{G}} m_{\mathbf{i}}^2\right)(1-\mathsf{s})\frac{\mathsf{f}'}{\mathsf{f}} \\
&= \mathsf{s}' + (1-\mathsf{s})\frac{\mathsf{f}'}{\mathsf{f}} - \frac{1}{N}\left(\mathsf{s}'m_{\mathbf{0}} + (1-\mathsf{s})\frac{\mathsf{f}'}{\mathsf{f}}\sum_{\mathbf{i}\in\mathbf{G}} m_{\mathbf{i}}^2\right).
\end{aligned} \tag{A-63}$$

Applying the same line of arguments produces

$$\frac{\partial \tilde{w}}{\partial \pi_{\mathbf{0}}} = -\frac{N-1}{N}\left[m_{\mathbf{0}}\mathsf{s}' + \left(\sum_{\mathbf{i}\in\mathbf{G}} m_{\mathbf{i}}^2\right)(1-\mathsf{s})\frac{\mathsf{f}'}{\mathsf{f}}\right] \tag{A-64}$$

and

$$\frac{\partial \tilde{w}}{\partial \pi_{\mathbf{j}}} = -m_{\mathbf{j}}\mathsf{s}' - \left(\sum_{\mathbf{i}\in\mathbf{G}} m_{\mathbf{i}}m_{\mathbf{i}-\mathbf{j}}\right)(1-\mathsf{s})\frac{\mathsf{f}'}{\mathsf{f}} \text{ for } \mathbf{j} \neq \mathbf{0}, \tag{A-65}$$

where, as usual, all functions are evaluated at the resident trait value z and equilibrium $\hat{n}$.

Introducing the notation

$$P_{\mathbf{j}} = \sum_{\mathbf{i}\in\mathbf{G}} m_{\mathbf{i}}m_{\mathbf{i}-\mathbf{j}}, \tag{A-66}$$

which is the probability that an offspring born in patch $\mathbf{j}$ competes with an offspring of the focal individual (i.e. that they both migrate to the same patch), the derivatives in eqs. (A-63)–(A-65) can be

more compactly written as

$$\frac{\partial \tilde{w}}{\partial \pi_\bullet} = \mathsf{s}' + (1-\mathsf{s})\frac{\mathsf{f}'}{\mathsf{f}} - \frac{1}{N}\left(\mathsf{s}' m_\mathbf{0} + (1-\mathsf{s})\frac{\mathsf{f}'}{\mathsf{f}} P_\mathbf{0}\right) \tag{A-67}$$

$$\frac{\partial \tilde{w}}{\partial \pi_\mathbf{0}} = -\frac{N-1}{N}\left(\mathsf{s}' m_\mathbf{0} + (1-\mathsf{s})\frac{\mathsf{f}'}{\mathsf{f}} P_\mathbf{0}\right) \tag{A-68}$$

$$\frac{\partial \tilde{w}}{\partial \pi_\mathbf{j}} = -\left(\mathsf{s}' m_\mathbf{j} + (1-\mathsf{s})\frac{\mathsf{f}'}{\mathsf{f}} P_\mathbf{j}\right) \text{ for } \mathbf{j} \neq \mathbf{0}. \tag{A-69}$$

In terms of these derivatives, the coefficients of fitness interdependence (A-41) can then be written as

$$\lambda_\mathbf{0} = -\frac{\partial \tilde{w}/\partial \pi_\mathbf{0}}{\partial \tilde{w}/\partial \pi_\bullet} = \left(\frac{N-1}{N}\right)\frac{\mathsf{s}'\mathsf{f} m_\mathbf{0} + \mathsf{f}'(1-\mathsf{s})P_\mathbf{0}}{\mathsf{s}'\mathsf{f} + \mathsf{f}'(1-\mathsf{s}) - \left[\mathsf{s}'\mathsf{f} m_\mathbf{0} + \mathsf{f}'(1-\mathsf{s})P_\mathbf{0}\right]/N}, \tag{A-70}$$

$$\lambda_\mathbf{j} = -\frac{\partial \tilde{w}/\partial \pi_\mathbf{j}}{\partial \tilde{w}/\partial \pi_\bullet} = \frac{\mathsf{s}'\mathsf{f} m_\mathbf{j} + \mathsf{f}'(1-\mathsf{s})P_\mathbf{j}}{\mathsf{s}'\mathsf{f} + \mathsf{f}'(1-\mathsf{s}) - \left[\mathsf{s}'\mathsf{f} m_\mathbf{0} + \mathsf{f}'(1-\mathsf{s})P_\mathbf{0}\right]/N} \text{ for } \mathbf{j} \neq \mathbf{0}. \tag{A-71}$$

Appendix E.2 Scaled-relatedness in terms of relatedness coefficients

To calculate and simplify the scaled-relatedness coefficients, it is convenient to start from expressions (A-55) – (A-57). First, note that using eqs. (A-67) and (A-68), and rearranging terms yields

$$\begin{aligned}
\frac{\partial \tilde{w}}{\partial \pi_\bullet} + \frac{\partial \tilde{w}}{\partial \pi_\mathbf{0}} R_{\mathbf{0},0} &= \mathsf{s}' + (1-\mathsf{s})\frac{\mathsf{f}'}{\mathsf{f}} - \left(\mathsf{s}' m_\mathbf{0} + (1-\mathsf{s})\frac{\mathsf{f}'}{\mathsf{f}} P_\mathbf{0}\right)\left(\frac{1}{N} + \frac{N-1}{N} R_{\mathbf{0},0}\right) \\
&= \mathsf{s}' + (1-\mathsf{s})\frac{\mathsf{f}'}{\mathsf{f}} - \left(\mathsf{s}' m_\mathbf{0} + (1-\mathsf{s})\frac{\mathsf{f}'}{\mathsf{f}} P_\mathbf{0}\right)\left(R_{\mathbf{0},0} + \frac{1}{N}\left(1 - R_{\mathbf{0},0}\right)\right) \\
&= \mathsf{s}' + (1-\mathsf{s})\frac{\mathsf{f}'}{\mathsf{f}} - \left(\mathsf{s}' m_\mathbf{0} + (1-\mathsf{s})\frac{\mathsf{f}'}{\mathsf{f}} P_\mathbf{0}\right) R_{\mathbf{0},0} \\
&\quad - \left(\mathsf{s}' m_\mathbf{0} + (1-\mathsf{s})\frac{\mathsf{f}'}{\mathsf{f}} P_\mathbf{0}\right)\frac{1}{N}\left(1 - R_{\mathbf{0},0}\right).
\end{aligned} \tag{A-72}$$

Then, using (A-72) and (A-69), rearranging, and factoring, the common denominator of $\kappa_{\mathbf{0},0}$ (A-55), $\kappa_{\mathbf{k},0}$ (A-56), and $\kappa_{\mathbf{k},t}$ (A-57) can be written as

$$\begin{aligned}
&\frac{\partial \tilde{w}}{\partial \pi_\bullet} + \frac{\partial \tilde{w}}{\partial \pi_\mathbf{0}} R_{\mathbf{0},0} + \sum_{\mathbf{j} \in \mathbb{G} \setminus \mathbf{0}} \frac{\partial \tilde{w}}{\partial \pi_\mathbf{j}} R_{\mathbf{j},0} \\
&= \mathsf{s}' + (1-\mathsf{s})\frac{\mathsf{f}'}{\mathsf{f}} - \left(\mathsf{s}' m_\mathbf{0} + (1-\mathsf{s})\frac{\mathsf{f}'}{\mathsf{f}} P_\mathbf{0}\right)\frac{1}{N}\left(1 - R_{\mathbf{0},0}\right) - \sum_{\mathbf{j} \in \mathbb{G}}\left(\mathsf{s}' m_\mathbf{j} + (1-\mathsf{s})\frac{\mathsf{f}'}{\mathsf{f}} P_\mathbf{j}\right) R_{\mathbf{j},0} \\
&= \mathsf{s}'\left[1 - \left(\sum_{\mathbf{j} \in \mathbb{G}} m_\mathbf{j} R_{\mathbf{j},0} + m_\mathbf{0}\frac{(1 - R_{\mathbf{0},0})}{N}\right)\right] + \frac{\mathsf{f}'}{\mathsf{f}}(1-\mathsf{s})\left[1 - \left(\sum_{\mathbf{j} \in \mathbb{G}} P_\mathbf{j} R_{\mathbf{j},0} + P_\mathbf{0}\frac{(1 - R_{\mathbf{0},0})}{N}\right)\right].
\end{aligned} \tag{A-73}$$

Similarly, using (A-68), yields

$$\begin{aligned}\frac{\partial\tilde{w}}{\partial\pi_{\mathbf{0}}}\left(\frac{1}{N-1}(1-R_{\mathbf{0},0})+R_{\mathbf{0},0}\right) &= -\frac{N-1}{N}\left(\mathsf{s}'m_{\mathbf{0}}+(1-\mathsf{s})\frac{\mathsf{f}'}{\mathsf{f}}P_{\mathbf{0}}\right)\left(\frac{1}{N-1}(1-R_{\mathbf{0},0})+R_{\mathbf{0},0}\right)\\ &= -\left(\mathsf{s}'m_{\mathbf{0}}+(1-\mathsf{s})\frac{\mathsf{f}'}{\mathsf{f}}P_{\mathbf{0}}\right)\left(\frac{1}{N}(1-R_{\mathbf{0},0})+\frac{N-1}{N}R_{\mathbf{0},0}\right)\\ &= -\left(\mathsf{s}'m_{\mathbf{0}}+(1-\mathsf{s})\frac{\mathsf{f}'}{\mathsf{f}}P_{\mathbf{0}}\right)\left(R_{\mathbf{0},0}-\frac{1}{N}R_{\mathbf{0},0}+\frac{1}{N}(1-R_{\mathbf{0},0})\right).\end{aligned}$$

Using this expression together with (A-67) and (A-69), the numerator of $\kappa_{\mathbf{0},0}$ (A-55) can be simplified as follows:

$$\begin{aligned}&R_{\mathbf{0},0}\frac{\partial\tilde{w}}{\partial\pi_{\bullet}}+\frac{\partial\tilde{w}}{\partial\pi_{\mathbf{0}}}\left(\frac{1}{N-1}(1-R_{\mathbf{0},0})+R_{\mathbf{0},0}\right)+\sum_{\mathbf{j}\in\mathbb{G}\setminus\mathbf{0}}\frac{\partial\tilde{w}}{\partial\pi_{\mathbf{j}}}R_{\mathbf{j},0}\\ &= R_{\mathbf{0},0}\left(\mathsf{s}'+(1-\mathsf{s})\frac{\mathsf{f}'}{\mathsf{f}}\right)-\frac{1}{N}\left(\mathsf{s}'m_{\mathbf{0}}+(1-\mathsf{s})\frac{\mathsf{f}'}{\mathsf{f}}P_{\mathbf{0}}\right)R_{\mathbf{0},0}\\ &-\left(\mathsf{s}'m_{\mathbf{0}}+(1-\mathsf{s})\frac{\mathsf{f}'}{\mathsf{f}}P_{\mathbf{0}}\right)\left(R_{\mathbf{0},0}-\frac{1}{N}R_{\mathbf{0},0}+\frac{1}{N}(1-R_{\mathbf{0},0})\right)-\sum_{\mathbf{j}\in\mathbb{G}\setminus\mathbf{0}}\left(\mathsf{s}'m_{\mathbf{j}}+(1-\mathsf{s})\frac{\mathsf{f}'}{\mathsf{f}}P_{\mathbf{j}}\right)R_{\mathbf{j},0},\\ &= R_{\mathbf{0},0}\left(\mathsf{s}'+(1-\mathsf{s})\frac{\mathsf{f}'}{\mathsf{f}}\right)-\left(\mathsf{s}'m_{\mathbf{0}}+(1-\mathsf{s})\frac{\mathsf{f}'}{\mathsf{f}}P_{\mathbf{0}}\right)\frac{1}{N}(1-R_{\mathbf{0},0})-\sum_{\mathbf{j}\in\mathbb{G}}\left(\mathsf{s}'m_{\mathbf{j}}+(1-\mathsf{s})\frac{\mathsf{f}'}{\mathsf{f}}P_{\mathbf{j}}\right)R_{\mathbf{j},0}\\ &= \mathsf{s}'\left[R_{\mathbf{0},0}-\left(\sum_{\mathbf{j}\in\mathbb{G}}m_{\mathbf{j}}R_{\mathbf{j},0}+m_{\mathbf{0}}\frac{(1-R_{\mathbf{0},0})}{N}\right)\right]+\frac{\mathsf{f}'}{\mathsf{f}}(1-\mathsf{s})\left[R_{\mathbf{0},0}-\left(\sum_{\mathbf{j}\in\mathbb{G}}P_{\mathbf{j}}R_{\mathbf{j},0}+P_{\mathbf{0}}\frac{(1-R_{\mathbf{0},0})}{N}\right)\right].\end{aligned}\tag{A-74}$$

Now, substituting (A-73) and (A-74) into (A-55), and then multiplying numerator and denominator by f, yields

$$\kappa_{\mathbf{0},0}=\frac{\mathsf{s}'\mathsf{f}\left[R_{\mathbf{0},0}-\left(\sum_{\mathbf{j}\in\mathbb{G}}m_{\mathbf{j}}R_{\mathbf{j},0}+m_{\mathbf{0}}\frac{(1-R_{\mathbf{0},0})}{N}\right)\right]+\mathsf{f}'(1-\mathsf{s})\left[R_{\mathbf{0},0}-\left(\sum_{\mathbf{j}\in\mathbb{G}}P_{\mathbf{j}}R_{\mathbf{j},0}+P_{\mathbf{0}}\frac{(1-R_{\mathbf{0},0})}{N}\right)\right]}{\mathsf{s}'\mathsf{f}\left[1-\left(\sum_{\mathbf{j}\in\mathbb{G}}m_{\mathbf{j}}R_{\mathbf{j},0}+m_{\mathbf{0}}\frac{(1-R_{\mathbf{0},0})}{N}\right)\right]+\mathsf{f}'(1-\mathsf{s})\left[1-\left(\sum_{\mathbf{j}\in\mathbb{G}}P_{\mathbf{j}}R_{\mathbf{j},0}+P_{\mathbf{0}}\frac{(1-R_{\mathbf{0},0})}{N}\right)\right]}.\tag{A-75}$$

To obtain similar expressions for $\kappa_{\mathbf{k},0}$ (A-56), and $\kappa_{\mathbf{k},t}$ (A-57), we first use eqs. (A-67)–(A-69) to write

$$
\begin{aligned}
& R_{\mathbf{k},t}\frac{\partial\tilde{w}}{\partial\pi_{\bullet}} + \sum_{\mathbf{j}\in\mathbb{G}}\frac{\partial\tilde{w}}{\partial\pi_{\mathbf{j}}}R_{\mathbf{j}-\mathbf{k},t} \\
&= R_{\mathbf{k},t}\frac{\partial\tilde{w}}{\partial\pi_{\bullet}} + R_{-\mathbf{k},t}\frac{\partial\tilde{w}}{\partial\pi_{\mathbf{0}}} + \sum_{\mathbf{j}\in\mathbb{G}\setminus\mathbf{0}}\frac{\partial\tilde{w}}{\partial\pi_{\mathbf{j}}}R_{\mathbf{j}-\mathbf{k},t} \\
&= R_{\mathbf{k},t}\left(\frac{\partial\tilde{w}}{\partial\pi_{\bullet}} + \frac{\partial\tilde{w}}{\partial\pi_{\mathbf{0}}}\right) + \sum_{\mathbf{j}\in\mathbb{G}\setminus\mathbf{0}}\frac{\partial\tilde{w}}{\partial\pi_{\mathbf{j}}}R_{\mathbf{j}-\mathbf{k},t} \\
&= R_{\mathbf{k},t}\left[\mathsf{s}' + (1-\mathsf{s})\frac{\mathsf{f}'}{\mathsf{f}} - \frac{1}{N}\left(\mathsf{s}'m_{\mathbf{0}} + (1-\mathsf{s})\frac{\mathsf{f}'}{\mathsf{f}}P_{\mathbf{0}}\right) - \frac{N-1}{N}\left(\mathsf{s}'m_{\mathbf{0}} + (1-\mathsf{s})\frac{\mathsf{f}'}{\mathsf{f}}P_{\mathbf{0}}\right)\right] + \sum_{\mathbf{j}\in\mathbb{G}\setminus\mathbf{0}}\frac{\partial\tilde{w}}{\partial\pi_{\mathbf{j}}}R_{\mathbf{j}-\mathbf{k},t} \\
&= R_{\mathbf{k},t}\left[\mathsf{s}' + (1-\mathsf{s})\frac{\mathsf{f}'}{\mathsf{f}} - \left(\mathsf{s}'m_{\mathbf{0}} + (1-\mathsf{s})\frac{\mathsf{f}'}{\mathsf{f}}P_{\mathbf{0}}\right)\right] - \sum_{\mathbf{j}\in\mathbb{G}\setminus\mathbf{0}}\left[\mathsf{s}'m_{\mathbf{j}} + (1-\mathsf{s})\frac{\mathsf{f}'}{\mathsf{f}}P_{\mathbf{j}}\right]R_{\mathbf{j}-\mathbf{k},t} \\
&= R_{\mathbf{k},t}\left[\mathsf{s}' + (1-\mathsf{s})\frac{\mathsf{f}'}{\mathsf{f}}\right] - \sum_{\mathbf{j}\in\mathbb{G}}\left[\mathsf{s}'m_{\mathbf{j}} + (1-\mathsf{s})\frac{\mathsf{f}'}{\mathsf{f}}P_{\mathbf{j}}\right]R_{\mathbf{j}-\mathbf{k},t} \\
&= \mathsf{s}'\left[R_{\mathbf{k},t} - \sum_{\mathbf{j}\in\mathbb{G}}m_{\mathbf{j}}R_{\mathbf{j}-\mathbf{k},t}\right] + \frac{\mathsf{f}'}{\mathsf{f}}(1-\mathsf{s})\left[R_{\mathbf{k},t} - \sum_{\mathbf{j}\in\mathbb{G}}P_{\mathbf{j}}R_{\mathbf{j}-\mathbf{k},t}\right]. \qquad \text{(A-76)}
\end{aligned}
$$

and

$$
\begin{aligned}
\frac{\partial\tilde{w}}{\partial\pi_{\mathbf{k}}}\frac{1}{N}(1-R_{\mathbf{k},0}) &= -\left(\mathsf{s}'m_{\mathbf{k}} + (1-\mathsf{s})\frac{\mathsf{f}'}{\mathsf{f}}P_{\mathbf{k}}\right)\frac{1}{N}(1-R_{\mathbf{k},0}) \\
&= -\mathsf{s}'m_{\mathbf{k}}\frac{(1-R_{\mathbf{k},0})}{N} - \frac{\mathsf{f}'(1-\mathsf{s})}{\mathsf{f}}P_{\mathbf{k}}\frac{(1-R_{\mathbf{k},0})}{N} \qquad \text{(A-77)}
\end{aligned}
$$

Substituting (A-73), (A-77), and (A-76) into (A-56), and then multiplying numerator and denominator by f, yields

$$
\kappa_{\mathbf{k},0} = \frac{\mathsf{s}'\mathsf{f}\left[R_{\mathbf{k},t} - \left(\sum_{\mathbf{j}\in\mathbb{G}}m_{\mathbf{j}}R_{\mathbf{j}-\mathbf{k},0} + m_{\mathbf{k}}\frac{(1-R_{\mathbf{0},0})}{N}\right)\right] + \mathsf{f}'(1-\mathsf{s})\left[R_{\mathbf{k},t} - \left(\sum_{\mathbf{j}\in\mathbb{G}}P_{\mathbf{j}}R_{\mathbf{j}-\mathbf{k},0} + P_{\mathbf{k}}\frac{(1-R_{\mathbf{0},0})}{N}\right)\right]}{\mathsf{s}'\mathsf{f}\left[1 - \left(\sum_{\mathbf{j}\in\mathbb{G}}m_{\mathbf{j}}R_{\mathbf{j},0} + m_{\mathbf{0}}\frac{(1-R_{\mathbf{0},0})}{N}\right)\right] + \mathsf{f}'(1-\mathsf{s})\left[1 - \left(\sum_{\mathbf{j}\in\mathbb{G}}P_{\mathbf{j}}R_{\mathbf{j},0} + P_{\mathbf{0}}\frac{(1-R_{\mathbf{0},0})}{N}\right)\right]} \text{ for } \mathbf{k} \neq \mathbf{0}. \qquad \text{(A-78)}
$$

Likewise, substituting (A-73), and (A-76) into (A-57), and then multiplying numerator and denominator by f, yields

$$
\kappa_{\mathbf{k},t} = \frac{\mathsf{s}'\mathsf{f}\left[R_{\mathbf{k},t} - \sum_{\mathbf{j}\in\mathbb{G}}m_{\mathbf{j}}R_{\mathbf{j}-\mathbf{k},t}\right] + \mathsf{f}'(1-\mathsf{s})\left[R_{\mathbf{k},t} - \sum_{\mathbf{j}\in\mathbb{G}}P_{\mathbf{j}}R_{\mathbf{j}-\mathbf{k},t}\right]}{\mathsf{s}'\mathsf{f}\left[1 - \left(\sum_{\mathbf{j}\in\mathbb{G}}m_{\mathbf{j}}R_{\mathbf{j},0} + m_{\mathbf{0}}\frac{(1-R_{\mathbf{0},0})}{N}\right)\right] + \mathsf{f}'(1-\mathsf{s})\left[1 - \left(\sum_{\mathbf{j}\in\mathbb{G}}P_{\mathbf{j}}R_{\mathbf{j},0} + P_{\mathbf{0}}\frac{(1-R_{\mathbf{0},0})}{N}\right)\right]} \text{ for } t > 0. \qquad \text{(A-79)}
$$

Appendix E.3 Scaled-relatedness in terms of demographic parameters

Substituting eq. (7) into eqs. (A-75), (A-78), and (A-79), and then cancelling common terms, we obtain

$$\kappa_{\mathbf{0},0} = \frac{\mathsf{s}'\mathsf{f} \lim_{\mu\to 0}\left[Q_{\mathbf{0},0} - \left(\sum_{\mathbf{j}\in\mathbb{G}} m_{\mathbf{j}} Q_{\mathbf{j},0} + m_{\mathbf{0}} \frac{(1-Q_{\mathbf{0},0})}{N}\right)\right] + \mathsf{f}'(1-\mathsf{s}) \lim_{\mu\to 0}\left[Q_{\mathbf{0},0} - \left(\sum_{\mathbf{j}\in\mathbb{G}} P_{\mathbf{j}} Q_{\mathbf{j},0} + P_{\mathbf{0}} \frac{(1-Q_{\mathbf{0},0})}{N}\right)\right]}{\mathsf{s}'\mathsf{f} \lim_{\mu\to 0}\left[1 - \left(\sum_{\mathbf{j}\in\mathbb{G}} m_{\mathbf{j}} Q_{\mathbf{j},0} + m_{\mathbf{0}} \frac{(1-Q_{\mathbf{0},0})}{N}\right)\right] + \mathsf{f}'(1-\mathsf{s}) \lim_{\mu\to 0}\left[1 - \left(\sum_{\mathbf{j}\in\mathbb{G}} P_{\mathbf{j}} Q_{\mathbf{j},0} + P_{\mathbf{0}} \frac{(1-Q_{\mathbf{0},0})}{N}\right)\right]}, \quad \text{(A-80)}$$

$$\kappa_{\mathbf{k},0} = \frac{\mathsf{s}'\mathsf{f} \lim_{\mu\to 0}\left[Q_{\mathbf{k},0} - \left(\sum_{\mathbf{j}\in\mathbb{G}} m_{\mathbf{j}} Q_{\mathbf{j}-\mathbf{k},0} + m_{\mathbf{k}} \frac{(1-Q_{\mathbf{0},0})}{N}\right)\right] + \mathsf{f}'(1-\mathsf{s}) \lim_{\mu\to 0}\left[Q_{\mathbf{k},0} - \left(\sum_{\mathbf{j}\in\mathbb{G}} P_{\mathbf{j}} Q_{\mathbf{j}-\mathbf{k},0} + P_{\mathbf{k}} \frac{(1-Q_{\mathbf{0},0})}{N}\right)\right]}{\mathsf{s}'\mathsf{f} \lim_{\mu\to 0}\left[1 - \left(\sum_{\mathbf{j}\in\mathbb{G}} m_{\mathbf{j}} Q_{\mathbf{j},0} + m_{\mathbf{0}} \frac{(1-Q_{\mathbf{0},0})}{N}\right)\right] + \mathsf{f}'(1-\mathsf{s}) \lim_{\mu\to 0}\left[1 - \left(\sum_{\mathbf{j}\in\mathbb{G}} P_{\mathbf{j}} Q_{\mathbf{j},0} + P_{\mathbf{0}} \frac{(1-Q_{\mathbf{0},0})}{N}\right)\right]}, \quad \text{(A-81)}$$

for $\mathbf{k} \neq \mathbf{0}$, and

$$\kappa_{\mathbf{k},t} = \frac{\mathsf{s}'\mathsf{f} \lim_{\mu\to 0}\left[Q_{\mathbf{k},t} - \sum_{\mathbf{j}\in\mathbb{G}} m_{\mathbf{j}} Q_{\mathbf{j}-\mathbf{k},t}\right] + \mathsf{f}'(1-\mathsf{s}) \lim_{\mu\to 0}\left[Q_{\mathbf{k},t} - \sum_{\mathbf{j}\in\mathbb{G}} P_{\mathbf{j}} Q_{\mathbf{j}-\mathbf{k},t}\right]}{\mathsf{s}'\mathsf{f} \lim_{\mu\to 0}\left[1 - \left(\sum_{\mathbf{j}\in\mathbb{G}} m_{\mathbf{j}} Q_{\mathbf{j},0} + m_{\mathbf{0}} \frac{(1-Q_{\mathbf{0},0})}{N}\right)\right] + \mathsf{f}'(1-\mathsf{s}) \lim_{\mu\to 0}\left[1 - \left(\sum_{\mathbf{j}\in\mathbb{G}} P_{\mathbf{j}} Q_{\mathbf{j},0} + P_{\mathbf{0}} \frac{(1-Q_{\mathbf{0},0})}{N}\right)\right]}. \quad \text{(A-82)}$$

for $t > 0$.

To simplify eqs. (A-80)–(A-82), we first note that, from eqs. (A.42), (A.48), and (A.51) in [8], we have[1]

$$\lim_{\mu\to 0} \frac{1}{1-Q_{\mathbf{0},0}}\left[1 - \left(\sum_{\mathbf{j}\in\mathbb{G}} m_{\mathbf{j}} Q_{\mathbf{j},0} + m_{\mathbf{0}} \frac{(1-Q_{\mathbf{0},0})}{N}\right)\right] = \frac{1}{N}\left[N + \mathcal{L}_{\mathbf{0}}\left(F^{\mathsf{s}}\right) - \frac{1+\mathsf{s}}{2D}\right], \quad \text{(A-83)}$$

$$\lim_{\mu\to 0} \frac{1}{1-Q_{\mathbf{0},0}}\left[Q_{\mathbf{0},0} - \left(\sum_{\mathbf{j}\in\mathbb{G}} m_{\mathbf{j}} Q_{\mathbf{j},0} + m_{\mathbf{0}} \frac{(1-Q_{\mathbf{0},0})}{N}\right)\right] = \frac{1}{N}\left[\mathcal{L}_{\mathbf{0}}\left(F^{\mathsf{s}}\right) - \frac{1+\mathsf{s}}{2D}\right], \quad \text{(A-84)}$$

$$\lim_{\mu\to 0} \frac{1}{1-Q_{\mathbf{0},0}}\left[Q_{\mathbf{k},0} - \left(\sum_{\mathbf{j}\in\mathbb{G}} m_{\mathbf{j}} Q_{\mathbf{j}-\mathbf{k},0} + m_{\mathbf{k}} \frac{(1-Q_{\mathbf{0},0})}{N}\right)\right] = \frac{1}{N}\left[\mathcal{L}_{\mathbf{k}}\left(F^{\mathsf{s}}\right) - \frac{1+\mathsf{s}}{2D}\right] \text{ for } \mathbf{k} \neq \mathbf{0}, \quad \text{(A-85)}$$

$$\lim_{\mu\to 0} \frac{1}{1-Q_{\mathbf{0},0}}\left[Q_{\mathbf{k},t} - \sum_{\mathbf{j}\in\mathbb{G}} m_{\mathbf{j}} Q_{\mathbf{j}-\mathbf{k},t}\right] = \frac{1}{N}\left[\mathcal{L}_{\mathbf{k}}\left(G^{\mathsf{s}}_t\right) - \frac{1+\mathsf{s}}{2D}\right] \text{ for } t \neq 0, \quad \text{(A-86)}$$

where $\mathcal{L}_{\mathbf{k}}(\mathcal{F})$ is the inverse transform of $\mathcal{F}$ at $\mathbf{k}$ as defined in eq. (I.B), and where the functions F^{s} and

[1] Eq. (A.51) in [8] applies for all $\mathbf{k} \in \mathbb{G}$, the condition " if $\mathbf{k} > \mathbf{0}$" therein is not necessary. Also, the term $-s/N$ in the last line of eq (A.48) of [8] contains a typo and should be replaced by s/N.

G_t^{s} are defined at $\mathbf{h}$ as

$$F^{\mathrm{s}}(\mathbf{h}) = -\frac{(1-\mathrm{s})\mathcal{M}(\mathbf{h})}{1+\mathrm{s}+(1-\mathrm{s})\mathcal{M}(\mathbf{h})}, \tag{A-87}$$

$$G_t^{\mathrm{s}}(\mathbf{h}) = \frac{(1+\mathrm{s})\left[\mathrm{s}+(1-\mathrm{s})\mathcal{M}(\mathbf{h})\right]^t}{1+\mathrm{s}+(1-\mathrm{s})\mathcal{M}(\mathbf{h})} \tag{A-88}$$

(see eqs. (A.47) and (A.49) in [8]).

Likewise, from eqs. (A.32), (A.38), and (A.41)[2] in [8], we have

$$\lim_{\mu\to 0}\frac{1}{1-Q_{\mathbf{0},0}}\left[1-\left(\sum_{\mathbf{j}\in\mathbb{G}}P_{\mathbf{j}}Q_{\mathbf{j},0}+P_{\mathbf{0}}\frac{(1-Q_{\mathbf{0},0})}{N}\right)\right]=\frac{1}{N}\left[N+\mathcal{L}_{\mathbf{0}}\left(F^{\mathrm{f}}\right)-\frac{1+\mathrm{s}}{D}\right] \tag{A-89}$$

$$\lim_{\mu\to 0}\frac{1}{1-Q_{\mathbf{0},0}}\left[Q_{\mathbf{0},0}-\left(\sum_{\mathbf{j}\in\mathbb{G}}P_{\mathbf{j}}Q_{\mathbf{j},0}+P_{\mathbf{0}}\frac{(1-Q_{\mathbf{0},0})}{N}\right)\right]=\frac{1}{N}\left[\mathcal{L}_{\mathbf{0}}\left(F^{\mathrm{f}}\right)-\frac{1+\mathrm{s}}{D}\right] \tag{A-90}$$

$$\lim_{\mu\to 0}\frac{1}{1-Q_{\mathbf{0},0}}\left[Q_{\mathbf{k},0}-\left(\sum_{\mathbf{j}\in\mathbb{G}}P_{\mathbf{j}}Q_{\mathbf{j}-\mathbf{k},0}+P_{\mathbf{k}}\frac{(1-Q_{\mathbf{0},0})}{N}\right)\right]=\frac{1}{N}\left[\mathcal{L}_{\mathbf{k}}\left(F^{\mathrm{f}}\right)-\frac{1+\mathrm{s}}{D}\right]\text{ for }\mathbf{k}\neq\mathbf{0}, \tag{A-91}$$

$$\lim_{\mu\to 0}\frac{1}{1-Q_{\mathbf{0},0}}\left[Q_{\mathbf{k},t}-\sum_{\mathbf{j}\in\mathbb{G}}P_{\mathbf{j}}Q_{\mathbf{j}-\mathbf{k},t}\right]=\frac{1}{N}\left[\mathcal{L}_{\mathbf{k}}\left(G_t^{\mathrm{f}}\right)-\frac{1+\mathrm{s}}{D}\right]\text{ for }t\neq 0, \tag{A-92}$$

where the functions F^{f} and G_t^{f} are defined at $\mathbf{h}$ as

$$F^{\mathrm{f}}(\mathbf{h}) = \frac{2\mathrm{s}\mathcal{M}(\mathbf{h})}{1+\mathrm{s}+(1-\mathrm{s})\mathcal{M}(\mathbf{h})}, \tag{A-93}$$

$$G_t^{\mathrm{f}}(\mathbf{h}) = \frac{(1+\mathrm{s})(1+\mathcal{M}(\mathbf{h}))\left[\mathrm{s}+(1-\mathrm{s})\mathcal{M}(\mathbf{h})\right]^t}{1+\mathrm{s}+(1-\mathrm{s})\mathcal{M}(\mathbf{h})} \tag{A-94}$$

(see eqs. (A.37) and (A.39)[3] in [8]).

We can now proceed to simplify eqs. (A-80)–(A-82). First, multiplying the numerator and denominator of (A-80) by $\lim_{\mu\to 0}1/(1-Q_{\mathbf{0},0})$, substituting eqs. (A-83), (A-84), (A-89), and (A-90), and then multiplying numerator and denominator by N, we obtain

$$\kappa_{\mathbf{0},0}=\frac{\mathrm{s}'\mathrm{f}\left[\mathcal{L}_{\mathbf{0}}\left(F^{\mathrm{s}}\right)-\frac{1+\mathrm{s}}{2D}\right]+\mathrm{f}'(1-\mathrm{s})\left[\mathcal{L}_{\mathbf{0}}\left(F^{\mathrm{f}}\right)-\frac{1+\mathrm{s}}{D}\right]}{\mathrm{s}'\mathrm{f}\left[N+\mathcal{L}_{\mathbf{0}}\left(F^{\mathrm{s}}\right)-\frac{1+\mathrm{s}}{2D}\right]+\mathrm{f}'(1-\mathrm{s})\left[N+\mathcal{L}_{\mathbf{0}}\left(F^{\mathrm{f}}\right)-\frac{1+\mathrm{s}}{D}\right]}. \tag{A-95}$$

Second, proceeding similarly with eq. (A-81) (by substituting eqs. (A-83), (A-85), (A-89), and (A-91)), we obtain

$$\kappa_{\mathbf{k},0}=\frac{\mathrm{s}'\mathrm{f}\left[\mathcal{L}_{\mathbf{k}}\left(F^{\mathrm{s}}\right)-\frac{1+\mathrm{s}}{2D}\right]+\mathrm{f}'(1-\mathrm{s})\left[\mathcal{L}_{\mathbf{k}}\left(F^{\mathrm{f}}\right)-\frac{1+\mathrm{s}}{D}\right]}{\mathrm{s}'\mathrm{f}\left[N+\mathcal{L}_{\mathbf{0}}\left(F^{\mathrm{s}}\right)-\frac{1+\mathrm{s}}{2D}\right]+\mathrm{f}'(1-\mathrm{s})\left[N+\mathcal{L}_{\mathbf{0}}\left(F^{\mathrm{f}}\right)-\frac{1+\mathrm{s}}{D}\right]}\text{ for }\mathbf{k}\neq\mathbf{0}. \tag{A-96}$$

[2] Eq. (A.41) in [8] applies for for all $\mathbf{k}\in\mathbb{G}$, the condition " if $\mathbf{k}\neq\mathbf{0}$" therein is not necessary.

[3] Eq.(A.39) in [8] contains a typo in that the the second parenthesis is not closed and the term $(1+\psi_{\mathbf{h}}$ should read $(1+\psi_{\mathbf{h}})$.

Third, proceeding similarly with eq. (A-82) with $t > 0$ (by substituting eqs. (A-83), (A-86), (A-89), and (A-92)) we obtain

$$\kappa_{\mathbf{k},t} = \frac{\mathsf{s}'\mathsf{f}\left[\mathcal{L}_{\mathbf{k}}\left(G_t^{\mathsf{s}}\right) - \frac{1+\mathsf{s}}{2D}\right] + \mathsf{f}'(1-\mathsf{s})\left[\mathcal{L}_{\mathbf{k}}\left(G^{\mathsf{f}}\right) - \frac{1+\mathsf{s}}{D}\right]}{\mathsf{s}'\mathsf{f}\left[N + \mathcal{L}_{\mathbf{0}}\left(F^{\mathsf{s}}\right) - \frac{1+\mathsf{s}}{2D}\right] + \mathsf{f}'(1-\mathsf{s})\left[N + \mathcal{L}_{\mathbf{0}}\left(F^{\mathsf{f}}\right) - \frac{1+\mathsf{s}}{D}\right]} \quad \text{for } t > 0. \tag{A-97}$$

Finally, noting that eq. (A-95) is equal to eq. (A-96) with $\mathbf{k} = \mathbf{0}$, substituting eqs. (A-87)–(A-93), and rearranging yields eq. (III.A) of Box 2, that is:

$$\kappa_{\mathbf{k},t} = \begin{cases} \dfrac{\mathcal{L}_{\mathbf{k}}(F) - (1+\mathsf{s})\left[\mathsf{s}'\mathsf{f} + 2\mathsf{f}'(1-\mathsf{s})\right]/(2D)}{N\left[\mathsf{s}'\mathsf{f} + \mathsf{f}'(1-\mathsf{s})\right] + \mathcal{L}_{\mathbf{0}}(F) - (1+\mathsf{s})\left[\mathsf{s}'\mathsf{f} + 2\mathsf{f}'(1-\mathsf{s})\right]/(2D)} & \text{if } t = 0 \\ \dfrac{\mathcal{L}_{\mathbf{k}}(G_t) - (1+\mathsf{s})\left[\mathsf{s}'\mathsf{f} + 2\mathsf{f}'(1-\mathsf{s})\right]/(2D)}{N\left[\mathsf{s}'\mathsf{f} + \mathsf{f}'(1-\mathsf{s})\right] + \mathcal{L}_{\mathbf{0}}(F) - (1+\mathsf{s})\left[\mathsf{s}'\mathsf{f} + 2\mathsf{f}'(1-\mathsf{s})\right]/(2D)} & \text{otherwise,} \end{cases} \tag{A-98}$$

where the functions F and G_t are given by

$$\begin{aligned} F(\mathbf{h}) &= -\frac{(1-\mathsf{s})\left[\mathsf{s}'\mathsf{f} - 2\mathsf{s}\mathsf{f}'\right]\mathcal{M}(\mathbf{h})}{1+\mathsf{s}+(1-\mathsf{s})\mathcal{M}(\mathbf{h})}, \\ G_t(\mathbf{h}) &= \frac{(1+\mathsf{s})\left[\mathsf{s}'\mathsf{f} + \mathsf{f}'(1-\mathsf{s})(1+\mathcal{M}(\mathbf{h}))\right]\left[\mathsf{s}+(1-\mathsf{s})\mathcal{M}(\mathbf{h})\right]^t}{1+\mathsf{s}+(1-\mathsf{s})\mathcal{M}(\mathbf{h})}, \end{aligned} \tag{A-99}$$

as required.

Note that one can also write eq. (A-98) as

$$\kappa_{\mathbf{k},t} = \begin{cases} \dfrac{\mathcal{L}_{\mathbf{k}}(F) - G_0(\mathbf{0})/D}{N\left[\mathsf{s}'\mathsf{f} + \mathsf{f}'(1-\mathsf{s})\right] + \mathcal{L}_{\mathbf{0}}(F) - G_0(\mathbf{0})/D} & \text{if } t = 0 \\ \dfrac{\mathcal{L}_{\mathbf{k}}(G_t) - G_0(\mathbf{0})/D}{N\left[\mathsf{s}'\mathsf{f} + \mathsf{f}'(1-\mathsf{s})\right] + \mathcal{L}_{\mathbf{0}}(F) - G_0(\mathbf{0})/D} & \text{otherwise,} \end{cases} \tag{A-100}$$

since, for all t,

$$G_t(\mathbf{0}) = \frac{(1+\mathsf{s})\left[\mathsf{s}'\mathsf{f} + \mathsf{f}'(1-\mathsf{s})(1+\mathcal{M}(\mathbf{0}))\right]\left[\mathsf{s}+(1-\mathsf{s})\mathcal{M}(\mathbf{0})\right]^t}{1+\mathsf{s}+(1-\mathsf{s})\mathcal{M}(\mathbf{0})} = G_0(\mathbf{0}) = \frac{(1+\mathsf{s})\left[\mathsf{s}'\mathsf{f} + 2\mathsf{f}'(1-\mathsf{s})\right]}{2} \tag{A-101}$$

holds.

Appendix E.4 Explicit expression for L

Finally, we evaluate L in eq. (A-47), which is needed if one aims to evaluate the trait stationary density function (A-5). Substituting the definition of the coefficients of fitness interdependence (A-41) into

eq. (A-47), simplifying, and rearranging, we obtain

$$L = \frac{\partial \tilde{w}}{\partial \pi_\bullet} + \frac{\partial \tilde{w}}{\partial \pi_{\mathbf{0}}} R_{\mathbf{0},0} + \sum_{\mathbf{j}\in\mathbb{G}\backslash\mathbf{0}} \frac{\partial \tilde{w}}{\partial \pi_{\mathbf{j}}} R_{\mathbf{j},0}$$
$$= \mathsf{s}' \left[1 - \left(\sum_{\mathbf{j}\in\mathbb{G}} m_{\mathbf{j}} R_{\mathbf{j},0} + m_{\mathbf{0}} \frac{(1-R_{\mathbf{0},0})}{N}\right)\right] + \frac{\mathsf{f}'}{\mathsf{f}}(1-\mathsf{s}) \left[1 - \left(\sum_{\mathbf{j}\in\mathbb{G}} P_{\mathbf{j}} R_{\mathbf{j},0} + P_{\mathbf{0}} \frac{(1-R_{\mathbf{0},0})}{N}\right)\right], \quad \text{(A-102)}$$

where the second equality follows from our previous derivation in eq. (A-73).

Substituting eq. (7), we get

$$L = \frac{1}{\mathsf{f}} \left(\mathsf{s}'\mathsf{f} \lim_{\mu\to 0} \frac{1}{1-\overline{Q}_0} \left[1 - \left(\sum_{\mathbf{j}\in\mathbb{G}} m_{\mathbf{j}} Q_{\mathbf{j},0} + m_{\mathbf{0}} \frac{(1-Q_{\mathbf{0},0})}{N}\right)\right] \right.$$
$$\left. + \mathsf{f}'(1-\mathsf{s}) \lim_{\mu\to 0} \frac{1}{1-\overline{Q}_0} \left[1 - \left(\sum_{\mathbf{j}\in\mathbb{G}} P_{\mathbf{j}} Q_{\mathbf{j},0} + P_{\mathbf{0}} \frac{(1-Q_{\mathbf{0},0})}{N}\right)\right]\right), \quad \text{(A-103)}$$

which can be computed as

$$L = \frac{1}{\mathsf{f}} \lim_{\mu\to 0} \left(\frac{1-Q_{\mathbf{0},0}}{1-\overline{Q}_0}\right) \left(\mathsf{s}'\mathsf{f} \lim_{\mu\to 0} \frac{1}{1-Q_{\mathbf{0},0}} \left[1 - \left(\sum_{\mathbf{j}\in\mathbb{G}} m_{\mathbf{j}} Q_{\mathbf{j},0} + m_{\mathbf{0}} \frac{(1-Q_{\mathbf{0},0})}{N}\right)\right] \right.$$
$$\left. + \mathsf{f}'(1-\mathsf{s}) \lim_{\mu\to 0} \frac{1}{1-Q_{\mathbf{0},0}} \left[1 - \left(\sum_{\mathbf{j}\in\mathbb{G}} P_{\mathbf{j}} Q_{\mathbf{j},0} + P_{\mathbf{0}} \frac{(1-Q_{\mathbf{0},0})}{N}\right)\right]\right). \quad \text{(A-104)}$$

Using eqs. (A-83), (A-89), and (A-99), this becomes after some rearrangements,

$$L = \lim_{\mu\to 0} \left(\frac{1-Q_{\mathbf{0},0}}{1-\overline{Q}_0}\right) \times \frac{1}{\mathsf{f}N} \left(N\left[\mathsf{s}'\mathsf{f} + \mathsf{f}'(1-\mathsf{s})\right] + \mathcal{L}_{\mathbf{0}}(F) - \frac{(1+\mathsf{s})\left[\mathsf{s}'\mathsf{f} + 2\mathsf{f}'(1-\mathsf{s})\right]}{2D}\right). \quad \text{(A-105)}$$

For a Wright-Fisher process where $\mathsf{s}' = \mathsf{s} = 0$ (and hence, also $\mathcal{L}_{\mathbf{0}}(F) = 0$), we have

$$L = \lim_{\mu\to 0} \left(\frac{1-Q_{\mathbf{0},0}}{1-\overline{Q}_0}\right) \times \frac{\mathsf{f}'}{\mathsf{f}N} \left(N - \frac{1}{D}\right) = \lim_{\mu\to 0} \left(\frac{1-Q_{\mathbf{0},0}}{1-\overline{Q}_0}\right) \times \frac{\mathsf{f}'}{\mathsf{f}} \left(\frac{ND-1}{ND}\right). \quad \text{(A-106)}$$

Appendix F Explicit coefficient for the selection gradient

Here, we derive eq. (III.C) of Box 3 of the main text. First, we simplify the expression for $\kappa_{\mathbf{k},t}$ for $t > 0$ given in the second line of eq. (A-100). Using the definition of the inverse Fourier transform given in

eq. (I.D), and simplifying we obtain

$$
\begin{aligned}
\kappa_{\mathbf{k},t} &= \frac{\mathcal{L}_{\mathbf{k}}(G_t) - G_t(\mathbf{0})/D}{N\left[\mathsf{s}'\mathsf{f} + \mathsf{f}'(1-\mathsf{s})\right] + \mathcal{L}_{\mathbf{0}}(F) - G_0(\mathbf{0})/D} \\
&= \frac{\frac{1}{D}\sum_{\mathbf{j}\in\mathbb{G}} G_t(\mathbf{j})\overline{\chi}_{\mathbf{k}}(\mathbf{j}) - \frac{1}{D}G_t(\mathbf{0})\overline{\chi}_{\mathbf{k}}(\mathbf{0})}{N\left[\mathsf{s}'\mathsf{f} + \mathsf{f}'(1-\mathsf{s})\right] + \frac{1}{D}\sum_{\mathbf{j}\in\mathbb{G}} F(\mathbf{j}) - G_0(\mathbf{0})/D} \\
&= \frac{\frac{1}{D}\sum_{\mathbf{j}\in\mathbb{G}\setminus\mathbf{0}} G_t(\mathbf{j})\overline{\chi}_{\mathbf{k}}(\mathbf{j})}{N\left[\mathsf{s}'\mathsf{f} + \mathsf{f}'(1-\mathsf{s})\right] + \frac{1}{D}\sum_{\mathbf{j}\in\mathbb{G}\setminus\mathbf{0}} F(\mathbf{j}) - \frac{\mathsf{s}'\mathsf{f}+\mathsf{f}'(1-\mathsf{s})}{D}} \\
&= \frac{\sum_{\mathbf{j}\in\mathbb{G}\setminus\mathbf{0}} G_t(\mathbf{j})\overline{\chi}_{\mathbf{k}}(\mathbf{j})}{(ND-1)\left[\mathsf{s}'\mathsf{f} + \mathsf{f}'(1-\mathsf{s})\right] + \sum_{\mathbf{j}\in\mathbb{G}\setminus\mathbf{0}} F(\mathbf{j})},
\end{aligned}
\tag{A-107}
$$

where we have used $\overline{\chi}_{\mathbf{k}}(\mathbf{0}) = 1$ for all $\mathbf{k} \in \mathbb{G}$, the identity $G_t(\mathbf{0}) = G_0(\mathbf{0})$ for all t (A-101), and the fact that

$$
F(\mathbf{0}) - G_0(\mathbf{0}) = -\left[\mathsf{s}'\mathsf{f} + \mathsf{f}'(1-\mathsf{s})\right] \tag{A-108}
$$

holds.

Substituting the simplified expression for $\kappa_{\mathbf{k},t}$ (A-107) together with the expression for $e_{\mathbf{k},t}$ (14) into eq. (24), and setting

$$
H = (ND-1)\left[\mathsf{s}'\mathsf{f} + \mathsf{f}'(1-\mathsf{s})\right] + \sum_{\mathbf{j}\in\mathbb{G}\setminus\mathbf{0}} F(\mathbf{j}), \tag{A-109}
$$

yields

$$
\begin{aligned}
K &= \sum_{t=1}^{\infty}\sum_{\mathbf{k}\in\mathbb{G}} e_{\mathbf{k},t}\kappa_{\mathbf{k},t} \\
&= \frac{1}{H}\sum_{t=1}^{\infty}\sum_{\mathbf{k}\in\mathbb{G}}\left[\frac{1}{D}\sum_{\mathbf{i}\in\mathbb{G}} \mathcal{C}(\mathbf{i})^{t-1}\Psi(\mathbf{i})\overline{\chi}_{\mathbf{k}}(\mathbf{i})\right]\left[\sum_{\mathbf{j}\in\mathbb{G}\setminus\mathbf{0}} G_t(\mathbf{j})\overline{\chi}_{\mathbf{k}}(\mathbf{j})\right] \\
&= \frac{1}{H}\sum_{t=1}^{\infty}\sum_{\mathbf{i}\in\mathbb{G}}\sum_{\mathbf{j}\in\mathbb{G}\setminus\mathbf{0}} \mathcal{C}(\mathbf{i})^{t-1}\Psi(\mathbf{i})G_t(\mathbf{j})\frac{1}{D}\sum_{\mathbf{k}\in\mathbb{G}}\overline{\chi}_{\mathbf{k}}(\mathbf{i})\overline{\chi}_{\mathbf{k}}(\mathbf{j}) \\
&= \frac{1}{H}\sum_{t=1}^{\infty}\sum_{\mathbf{j}\in\mathbb{G}\setminus\mathbf{0}} \mathcal{C}(-\mathbf{j})^{t-1}\Psi(-\mathbf{j})G_t(\mathbf{j}),
\end{aligned}
\tag{A-110}
$$

where the last equality follows from using eq. (I.F). Substituting eq. (III.B) into (A-110) and solving the geometric series yields

$$
\begin{aligned}
K &= \frac{1}{H}\sum_{t=1}^{\infty}\sum_{\mathbf{j}\in\mathbb{G}\setminus\mathbf{0}} \mathcal{C}(-\mathbf{j})^{t-1}\Psi(-\mathbf{j})\frac{(1+\mathsf{s})\left[\mathsf{s}'\mathsf{f} + \mathsf{f}'(1-\mathsf{s})(1+\mathcal{M}(\mathbf{j}))\right]\left[\mathsf{s} + (1-\mathsf{s})\mathcal{M}(\mathbf{j})\right]^t}{1+\mathsf{s}+(1-\mathsf{s})\mathcal{M}(\mathbf{j})} \\
&= \frac{1}{H}\sum_{\mathbf{j}\in\mathbb{G}\setminus\mathbf{0}} \frac{(1+\mathsf{s})\left[\mathsf{s}'\mathsf{f} + \mathsf{f}'(1-\mathsf{s})(1+\mathcal{M}(\mathbf{j}))\right]\left[\mathsf{s} + (1-\mathsf{s})\mathcal{M}(\mathbf{j}) - \mathcal{C}(-\mathbf{j})\mathcal{M}(\mathbf{j})\right]\Psi(-\mathbf{j})}{\left[1+\mathsf{s}+(1-\mathsf{s})\mathcal{M}(\mathbf{j})\right]\left[1-\mathcal{C}(-\mathbf{j})\right]\left[1-\mathcal{C}(-\mathbf{j})\mathcal{M}(\mathbf{j})\right]},
\end{aligned}
\tag{A-111}
$$

which is the final expression presented in eq. (III.C).

To go from the first to the second line of eq. (A-111), the relevant geometric series must converge,

which happens if the moduli of $\mathcal{M}(\mathbf{j})$ and $\mathcal{C}(\mathbf{j})$ are smaller than one (i.e. $|\mathcal{M}(\mathbf{j})| < 1$ and $|\mathcal{C}(\mathbf{j})| < 1$) for all $\mathbf{j} \neq \mathbf{0}$, i.e. if the complex numbers $\mathcal{M}(\mathbf{j})$ and $\mathcal{C}(\mathbf{j})$ are within the unit circle. To see this is true, consider first that by the property of characteristic functions of probability distributions, we have $\mathcal{M}(\mathbf{0}) = 1$, and $|\mathcal{M}(\mathbf{j})| < 1$ for $\mathbf{j} \neq \mathbf{0}$ (p. 182 in [9]). Second, $|\mathcal{C}(\mathbf{j})| < 1$ from our assumption that the dynamical system eq. (2) has a hyperbolically stable equilibrium point. Indeed, stability means that all the eigenvalues of the Jacobian matrix of eq. (2) have modulus smaller than one (e.g. p. 103 of [10]). But these eigenvalues are in fact given by the coefficients $\mathcal{C}(\mathbf{j})$. To see this, first note that from eq. (2) the Jacobian of this discrete-time dynamical system around the equilibrium $\hat{n}$ defined by eq. (3) is given by

$$\mathbf{J} = \begin{pmatrix} \frac{\partial g(\mathbf{z}_{0,t},\mathbf{n}_{0,t})}{\partial n_{0}} & \frac{\partial g(\mathbf{z}_{0,t},\mathbf{n}_{0,t})}{\partial n_{1}} & \frac{\partial g(\mathbf{z}_{0,t},\mathbf{n}_{0,t})}{\partial n_{2}} & \cdots & \frac{\partial g(\mathbf{z}_{0,t},\mathbf{n}_{0,t})}{\partial n_{\mathbf{D}_{-1}}} \\ \frac{\partial g(\mathbf{z}_{1,t},\mathbf{n}_{1,t})}{\partial n_{0}} & \frac{\partial g(\mathbf{z}_{1,t},\mathbf{n}_{1,t})}{\partial n_{1}} & \frac{\partial g(\mathbf{z}_{1,t},\mathbf{n}_{1,t})}{\partial n_{2}} & \cdots & \frac{\partial g(\mathbf{z}_{1,t},\mathbf{n}_{1,t})}{\partial n_{\mathbf{D}_{-1}}} \\ \frac{\partial g(\mathbf{z}_{2,t},\mathbf{n}_{2,t})}{\partial n_{0}} & \frac{\partial g(\mathbf{z}_{2,t},\mathbf{n}_{2,t})}{\partial n_{1}} & \frac{\partial g(\mathbf{z}_{2,t},\mathbf{n}_{2,t})}{\partial n_{2}} & \cdots & \frac{\partial g(\mathbf{z}_{2,t},\mathbf{n}_{2,t})}{\partial n_{\mathbf{D}_{-1}}} \\ \vdots & \vdots & \vdots & \ddots & \vdots \\ \frac{\partial g(\mathbf{z}_{\mathbf{D}_{-1},t},\mathbf{n}_{\mathbf{D}_{-1},t})}{\partial n_{0}} & \frac{\partial g(\mathbf{z}_{\mathbf{D}_{-1},t},\mathbf{n}_{\mathbf{D}_{-1},t})}{\partial n_{1}} & \frac{\partial g(\mathbf{z}_{\mathbf{D}_{-1},t},\mathbf{n}_{\mathbf{D}_{-1},t})}{\partial n_{2}} & \cdots & \frac{\partial g(\mathbf{z}_{\mathbf{D}_{-1},t},\mathbf{n}_{\mathbf{D}_{-1},t})}{\partial n_{\mathbf{D}_{-1}}} \end{pmatrix}, \tag{A-112}$$

where all derivatives are evaluated at z and $\hat{n}$. Now, recalling the notations defined in eq. (A-20), the entries of this matrix are of the form

$$c_{\mathbf{k}-\mathbf{i}} = \frac{\partial g(\mathbf{z}_{\mathbf{k},t},\mathbf{n}_{\mathbf{k},t})}{\partial n_{\mathbf{i},t}} = \frac{\partial g(\mathbf{z}_{\mathbf{k}-\mathbf{i},t},\mathbf{n}_{\mathbf{k}-\mathbf{i},t})}{\partial n_{\mathbf{0},t}} = \frac{\partial g(\mathbf{z}_{0,t},\mathbf{n}_{0,t})}{\partial n_{\mathbf{k}-\mathbf{i},t}}, \tag{A-113}$$

which is the same as eq. (A-20) since all phenotypes vectors, here and there, are set to $(z, ..., z)$ when computing the derivative. From the first equality in the previous equation, the Jacobian (A-112) can be written as

$$\mathbf{J} = \begin{pmatrix} c_{0} & c_{-1} & c_{-2} & \cdots \\ c_{1} & c_{0} & c_{-1} & \cdots \\ \vdots & \vdots & \vdots & \cdots \\ c_{D_{-1}} & c_{D_{-2}} & c_{D_{-3}} & \ddots \end{pmatrix}, \tag{A-114}$$

where we defined $\boldsymbol{D}_{-2} = \mathbf{D}_{-1} - \mathbf{1}$, $\boldsymbol{D}_{-3} = \mathbf{D}_{-1} - \mathbf{2}$, etc. Written in this form, it is clear that the Jacobian (A-114) is a $\mathbb{G}$-group circulant matrix (e.g. p. 50 of [11]), with eigenvalues given by the Fourier transform of $c_{\mathbf{j}}$ (Theorem 8 in [11]). Hence, the $\mathbf{k}$-th eigenvalue of $\mathbf{J}$ is $\mathcal{C}(\mathbf{k}) = \sum_{\mathbf{j}\in\mathbb{G}} c_{\mathbf{j}}\chi_{\mathbf{j}}(\mathbf{k})$.

Appendix G Public good diffusion example

Appendix G.1 Fecundity effects

Here, we derive eq. (33) of the main text, which considers fecundity effects and no generational overlap ($s' = s = 0$). Substituting eq. (21) into eq. (31) yields

$$\begin{aligned}\Omega &= \epsilon \sum_{t=1}^{\infty} \sum_{\mathbf{k}\in\mathbb{G}} (1-\epsilon)^{t-1} q_{\mathbf{k},t} \left(\frac{D p_{\mathbf{k},t} - 1}{DN - 1} \right) \\ &= \frac{\epsilon}{DN-1} \sum_{t=1}^{\infty} (1-\epsilon)^{t-1} \left(D \sum_{\mathbf{k}\in\mathbb{G}} q_{\mathbf{k},t} p_{\mathbf{k},t} - \sum_{\mathbf{k}\in\mathbb{G}} q_{\mathbf{k},t} \right) \\ &= \frac{\epsilon D}{DN-1} \sum_{t=1}^{\infty} (1-\epsilon)^{t-1} \left(\sum_{\mathbf{k}\in\mathbb{G}} p_{\mathbf{k},t} q_{\mathbf{k},t} - \frac{1}{D} \right), \end{aligned} \tag{A-115}$$

where we have used the fact that $q_{\mathbf{k},t}$ is a probability distribution over $\mathbb{G}$ for all t and hence that $\sum_{\mathbf{k}\in\mathbb{G}} q_{\mathbf{k},t} = 1$ holds for all t.

Eq. (A-115) can be written in terms of the population covariance of $p_{\mathbf{k},t}$ and $q_{\mathbf{k},t}$ in the following way. Recall that the population covariance of two vectors $x = (x_1, \dots, x_n)$ and $y = (y_1, \dots, y_n)$ of length n is given by

$$\text{cov}(x, y) = \frac{1}{n} \sum_{j=1}^{n} x_j y_j - \langle x \rangle \langle y \rangle \tag{A-116}$$

where $\langle x \rangle = (1/n) \sum_{j=1}^{n} x_j$ and $\langle y \rangle = (1/n) \sum_{j=1}^{n} y_j$. Using this definition of population covariance, and denoting by $p_t = (p_{\mathbf{0},t}, \dots, p_{\mathbf{D}_{-1},t})$ and $q_t = (q_{\mathbf{0},t}, \dots, q_{\mathbf{D}_{-1},t})$ the vectors collecting all $p_{\mathbf{k},t}$'s and $q_{\mathbf{k},t}$'s in lexicographic order, we can write

$$\begin{aligned}\text{cov}(p_t, q_t) &= \frac{1}{D} \sum_{\mathbf{k}\in\mathbb{G}} p_{\mathbf{k},t} q_{\mathbf{k},t} - \left(\frac{1}{D} \sum_{\mathbf{k}\in\mathbb{G}} p_{\mathbf{k},t} \right) \left(\frac{1}{D} \sum_{\mathbf{k}\in\mathbb{G}} q_{\mathbf{k},t} \right) \\ &= \frac{1}{D} \sum_{\mathbf{k}\in\mathbb{G}} p_{\mathbf{k},t} q_{\mathbf{k},t} - \frac{1}{D^2} \\ &= \frac{1}{D} \left(\sum_{\mathbf{k}\in\mathbb{G}} p_{\mathbf{k},t} q_{\mathbf{k},t} - \frac{1}{D} \right) \\ D\text{cov}(p_t, q_t) &= \sum_{\mathbf{k}\in\mathbb{G}} p_{\mathbf{k},t} q_{\mathbf{k},t} - \frac{1}{D}, \end{aligned} \tag{A-117}$$

where the second line follows from the fact that both $p_{\mathbf{k},t}$ and $q_{\mathbf{k},t}$ are probability distributions over $\mathbb{G}$ for all t and hence satisfy $\sum_{\mathbf{k}\in\mathbb{G}} p_{\mathbf{k},t} = \sum_{\mathbf{k}\in\mathbb{G}} q_{\mathbf{k},t} = 1$ for all t. Substituting (A-117) into (A-115) we finally obtain

$$\Omega = \frac{\epsilon D^2}{DN-1} \sum_{t=1}^{\infty} (1-\epsilon)^{t-1} \text{cov}(p_t, q_t), \tag{A-118}$$

as required.

Appendix G.2 Fecundity effects: Weak dispersal

Here, we derive eq. (35) of the main text, following the common approach to evaluate a weak migration approximation (chapter 3 in [2]). To do so, we first set $m_{\mathbf{0}} = (1-m)$ and $d_{\mathbf{0}} = (1-d)$, where m and d are the net dispersal probabilities of the focal species and the environmental variable, and write $m_{\mathbf{i}} = mg^{\mathrm{m}}_{\mathbf{i}}$ and $d_{\mathbf{i}} = dg^{\mathrm{d}}_{\mathbf{i}}$. The characteristic functions of the dispersal distributions can then be expressed as $\mathcal{M}(\mathbf{j}) = 1 - mx^{\mathrm{m}}(\mathbf{j})$ and $\mathcal{D}(\mathbf{j}) = 1 - dx^{\mathrm{d}}(\mathbf{j})$, where $x^{\mathrm{m}}(\mathbf{j}) = 1 - \sum_{\mathbf{i}\in\mathbb{G}\setminus\mathbf{0}} g^{\mathrm{m}}_{\mathbf{i}}\chi_{\mathbf{i}}(\mathbf{j})$ and $x^{\mathrm{d}}(\mathbf{j}) = 1 - \sum_{\mathbf{i}\in\mathbb{G}\setminus\mathbf{0}} g^{\mathrm{d}}_{\mathbf{i}}\chi_{\mathbf{i}}(\mathbf{j})$. Substituting these expressions into the summand of eq. (34), and Taylor expanding around $m = 0$ and $d = 0$, we get

$$\frac{\mathcal{D}(-\mathbf{j})\mathcal{M}(\mathbf{j})}{1-(1-\epsilon)\mathcal{D}(-\mathbf{j})\mathcal{M}(\mathbf{j})} = \frac{\epsilon - mx^{\mathrm{m}}(\mathbf{j}) - dx^{\mathrm{d}}(\mathbf{j})}{\epsilon^2} + \text{h.o.t.}, \tag{A-119}$$

where "h.o.t." refers to higher order terms, e.g. terms proportional to m^2, md, d^2, etc. Substituting $x^{\mathrm{m}}(\mathbf{j}) = [1-\mathcal{M}(\mathbf{j})]/m$ and $x^{\mathrm{d}}(\mathbf{j}) = [1-\mathcal{D}(\mathbf{j})]/d$, we can write eq. (34) as

$$\Omega = \frac{1}{DN-1}\sum_{\mathbf{j}\in\mathbb{G}\setminus\mathbf{0}} \frac{\mathcal{M}(\mathbf{j}) + \mathcal{D}(\mathbf{j}) + \epsilon - 2}{\epsilon^2} + \text{h.o.t.}. \tag{A-120}$$

Neglecting the higher order terms and using $\mathcal{M}(\mathbf{j}) = \sum_{\mathbf{k}\in\mathbb{G}} m_{\mathbf{k}}\chi_{\mathbf{k}}(\mathbf{j})$, and $\mathcal{D}(\mathbf{j}) = \sum_{\mathbf{k}\in\mathbb{G}} d_{\mathbf{k}}\chi_{\mathbf{k}}(\mathbf{j})$ produces

$$\begin{aligned}
\Omega &= \frac{1}{DN-1}\left[\sum_{\mathbf{j}\in\mathbb{G}\setminus\mathbf{0}}\sum_{\mathbf{k}\in\mathbb{G}}\left(\frac{m_{\mathbf{k}}+d_{\mathbf{k}}}{\epsilon^2}\right)\chi_{\mathbf{k}}(\mathbf{j}) + \sum_{\mathbf{j}\in\mathbb{G}\setminus\mathbf{0}}\left(\frac{\epsilon-2}{\epsilon^2}\right)\right] \qquad \text{(A-121)}\\
&= \frac{1}{DN-1}\left[\sum_{\mathbf{k}\in\mathbb{G}}\left(\frac{m_{\mathbf{k}}+d_{\mathbf{k}}}{\epsilon^2}\right)\left(\sum_{\mathbf{j}\in\mathbb{G}}\chi_{\mathbf{k}}(\mathbf{j}) - 1\right) + \sum_{\mathbf{j}\in\mathbb{G}\setminus\mathbf{0}}\left(\frac{\epsilon-2}{\epsilon^2}\right)\right]\\
&= \frac{1}{DN-1}\left[\left(\frac{m_{\mathbf{0}}+d_{\mathbf{0}}}{\epsilon^2}\right)(D-1) + (D-1)\left(\frac{\epsilon-2}{\epsilon^2}\right)\right]\\
&= \left(\frac{D-1}{DN-1}\right)\left(\frac{\epsilon-m-d}{\epsilon^2}\right),
\end{aligned}$$

where the penultimate equality follows from the facts that $\sum_{\mathbf{k}\in\mathbb{G}}\chi_{\mathbf{k}}(\mathbf{j}) = D$ if $\mathbf{j} = \mathbf{0}$ and zero otherwise (recall eq. I.F), and that $\sum_{\mathbf{j}\in\mathbb{G}\setminus\mathbf{0}} 1 = D - 1$.

Appendix G.3 Fecundity effects: Island model

Here, we derive the expression for Ω for a Wright-Fisher life cycle (using eq. 34) under the island model of species dispersal and commons movement, which we use in Fig 6 (dashed lines). Under this

model of dispersal and movement, we have

$$m_{\mathbf{k}} = \begin{cases} 1-m & \text{if } \mathbf{k} = \mathbf{0} \\ \dfrac{m}{D-1} & \text{otherwise,} \end{cases} \tag{A-122}$$

and similarly,

$$d_{\mathbf{k}} = \begin{cases} 1-d & \text{if } \mathbf{k} = \mathbf{0} \\ \dfrac{d}{D-1} & \text{otherwise.} \end{cases} \tag{A-123}$$

We first calculate the Fourier transforms $\mathcal{M}(\mathbf{h})$ and $\mathcal{D}(-\mathbf{h})$ of the dispersal and movement distributions. We obtain

$$\begin{aligned} \mathcal{M}(\mathbf{h}) &= \sum_{\mathbf{k}\in\mathbb{G}} m_{\mathbf{k}} \chi_{\mathbf{k}}(\mathbf{h}) \\ &= (1-m)\chi_{\mathbf{0}}(\mathbf{h}) + \sum_{\mathbf{k}\in\mathbb{G}\setminus\mathbf{0}} \frac{m}{D-1}\chi_{\mathbf{k}}(\mathbf{h}) \\ &= (1-m)\chi_{\mathbf{0}}(\mathbf{h}) + \frac{m}{D-1}\left[\sum_{\mathbf{k}\in\mathbb{G}} \chi_{\mathbf{k}}(\mathbf{h}) - \chi_{\mathbf{0}}(\mathbf{h})\right] \\ &= (1-m) + \frac{m}{D-1}\left[\sum_{\mathbf{k}\in\mathbb{G}} \chi_{\mathbf{k}}(\mathbf{h}) - 1\right] \\ &= \begin{cases} 1 & \text{if } \mathbf{h} = \mathbf{0} \\ \dfrac{(1-m)D-1}{D-1} & \text{otherwise,} \end{cases} \end{aligned} \tag{A-124}$$

where the first line uses the definition of Fourier transform (I.B), the second and third lines rearrange terms, the fourth line uses the fact that $\chi_{\mathbf{0}}(\mathbf{h}) = 0$, and the fifth line follows from identity (I.F). Likewise,

$$\mathcal{D}(\mathbf{h}) = \begin{cases} 1 & \text{if } \mathbf{h} = \mathbf{0} \\ \dfrac{(1-d)D-1}{D-1} & \text{otherwise.} \end{cases} \tag{A-125}$$

Substituting eqs. (A-124) and (A-125) into eq. (34), using the fact that, for (A-125) $\mathcal{D}(-\mathbf{h}) = \mathcal{D}(\mathbf{h})$ holds, and simplifying, we arrive to

$$\Omega = \left(\frac{\epsilon}{DN-1}\right) \frac{(D-1)\left[(1-d)D-1\right]\left[(1-m)D-1\right]}{(D-1)^2 - (1-\epsilon)\left[(1-d)D-1\right]\left[(1-m)D-1\right]}. \tag{A-126}$$

Appendix G.4 Survival effects

We now consider the case where there are survival but no fecundity effects ($\mathrm{f}' = 0$). Writing Ω as $\Omega = \epsilon KN/P'(z)$, substituting

$$\Psi(\mathbf{h}) = P'(z)\mathcal{D}(\mathbf{h})/N, \tag{A-127}$$

$$\mathcal{C}(\mathbf{h}) = (1-\epsilon)\mathcal{D}(\mathbf{h}). \tag{A-128}$$

into eq. (III.F) in Box 3, and simplifying we obtain

$$\begin{aligned}\Omega = &\frac{1}{(DN-1) - \sum_{\mathbf{j}\in\mathrm{G}\setminus\mathbf{0}} \frac{(1-\mathrm{s})\mathcal{M}(\mathbf{j})}{1+\mathrm{s}+(1-\mathrm{s})\mathcal{M}(\mathbf{j})}} \\ &\times \sum_{\mathbf{j}\in\mathrm{G}\setminus\mathbf{0}} \frac{(1+\mathrm{s})\left[\mathrm{s}+(1-\mathrm{s})\mathcal{M}(\mathbf{j}) - (1-\epsilon)\mathcal{M}(\mathbf{j})\mathcal{D}(-\mathbf{j})\right]\mathcal{D}(-\mathbf{j})}{\left[1+\mathrm{s}+(1-\mathrm{s})\mathcal{M}(\mathbf{j})\right]\left[1-(1-\epsilon)\mathcal{D}(-\mathbf{j})\right]\left[1-(1-\epsilon)\mathcal{M}(\mathbf{j})\mathcal{D}(-\mathbf{j})\right]},\end{aligned} \tag{A-129}$$

which remains a somewhat complicated expression. In the limit $\mathrm{s} \to 1$, eq. (A-129) simplifies to

$$\Omega = \frac{1}{DN-1} \sum_{\mathbf{j}\in\mathrm{G}\setminus\mathbf{0}} \frac{\mathcal{D}(-\mathbf{j})}{1-(1-\epsilon)\mathcal{D}(-\mathbf{j})}. \tag{A-130}$$

This can be thought of as a special case where investment into the common-pool resource occurs in a population of immortal individuals that, therefore, become mortal through endogenously induced deaths. Finally, we note that for a spatially symmetric dispersal distribution we can set $\mathcal{D}(-\mathbf{j}) = \mathcal{D}(\mathbf{j})$.

Appendix G.5 Species dispersal and commons movement

In our example, we assumed that the evolving species dispersed according to a model based on the binomial distribution, which is detailed in Appendix B. Hence, the characteristic function used for a one-dimensional habitat is given by eq. (A-10), while for a two-dimensional habitat, it is based on Appendix B.2.

We assume that the way the commons moves in space follows the same model as the evolving species. We write d for the commons' probability of movement (instead of m), and $\overline{\lambda}_{\mathrm{d}}$ for the mean number of steps a unit of commons moves conditional on leaving the patch (instead of $\overline{\lambda}_{\mathrm{m}}$). The characteristic function of the movement in one dimension then is like eq. (A-10), i.e.

$$\mathcal{D}(k) = (1-d) + d\sum_{j=1}^{\frac{D-1}{2}} p_j((D-1)/2, 2\lambda_{\mathrm{d}}/(D-1))\cos(2\pi jk/D) \tag{A-131}$$

where λ_{d} is such that

$$\overline{\lambda}_{\mathrm{d}} = \frac{\lambda_{\mathrm{d}}}{1 - \left(1 - \frac{2\lambda_{\mathrm{d}}}{D-1}\right)^{(D-1)/2}}. \tag{A-132}$$

Appendix G.6 Stationary distribution

Here, we specify the stationary distribution of the trait substitution sequence for our example, i.e. we specify eq. (A-5), which we used in Fig 6B for the interval. Substituting eq. (30) into eq. (A-54), which is in turn substituted into eq. (A-4) gives

$$\phi(z) = \underbrace{\lim_{\mu\to 0}\left(\frac{1-\overline{Q}_0}{1-Q_{\mathbf{0},0}}\right)}_{>0} \times L \times \pi(z,z,\hat{n}) \times \left(BP'(z)\alpha_{\mathrm{B}}\left(\frac{P(z)}{\epsilon}\right)^{\alpha_{\mathrm{B}}-1}\Omega - C\alpha_C z^{\alpha_{\mathrm{C}}-1}\right), \tag{A-133}$$

thus characterising the term within parenthesis of eq. (A-5). For the Wright-Fisher process, we have from eq. (A-106) that

$$L = \lim_{\mu\to 0}\left(\frac{1-Q_{\mathbf{0},0}}{1-\overline{Q}_0}\right) \times \frac{1}{\pi(z,z,\hat{n})}\left(\frac{DN-1}{DN}\right), \tag{A-134}$$

where we used the fact that, for our example, payoff is fecundity, and so $\mathrm{f}' = 1$ in eq. (A-106). Thus, the perturbation of the fixation probability reduces to

$$\phi(z) = \left(\frac{DN-1}{DN}\right)\left(BP'(z)\alpha_{\mathrm{B}}\left(\frac{P(z)}{\epsilon}\right)^{\alpha_{\mathrm{B}}-1}\Omega - C\alpha_C z^{\alpha_{\mathrm{C}}-1}\right). \tag{A-135}$$

Assuming further that $P(z) = P_0 z$, we find by substituting eq. (A-135) into eq. (A-5) that the stationary distribution is given by

$$p(z) = C_{\mathrm{p}} \exp\left[2\,(DN-1)\left(B\left(\frac{P_0 z}{\epsilon}\right)^{\alpha_{\mathrm{B}}}\epsilon\,\Omega - Cz^{\alpha_{\mathrm{C}}}\right)\right], \tag{A-136}$$

where C_{p} is a constant of proportionality such that $\int_{-\infty}^{\infty} p(z)\,\mathrm{d}z = 1$.

References

1. Rousset F, Billiard S. A theoretical basis for measures of kin selection in subdivided populations: finite populations and localized dispersal. Journal of Evolutionary Biology. 2000;13:814–825.
2. Rousset F. Genetic Structure and Selection in Subdivided Populations. Princeton, NJ: Princeton University Press; 2004.
3. Van Cleve J. Social evolution and genetic interactions in the short and long term. Theoretical Population Biology. 2015;103:2–26.
4. Lehmann L. Space-time relatedness and Hamilton's rule for long-lasting behaviors in viscous populations. American Naturalist. 2010;175(1):136–143.

5. Lehmann L. The evolution of trans-generational altruism: kin selection meets niche construction. Journal of Evolutionary Biology. 2007;20:181–189.

6. Lehmann L. The stationary distribution of a continuously varying strategy in a class-structured population under mutation-selection-drift balance. Journal of Evolutionary Biology. 2012;25:770–787.

7. Alger I, Weibull JW, Lehmann L. Evolution of preferences in structured populations: Genes, guns, and culture. Journal of Economic Theory. 2020;185:1–45.

8. Lehmann L, Rousset F. The evolution of social discounting in hierarchically clustered populations. Molecular Ecology. 2012;21:447–471.

9. Grimmett G, Stirzaker D. Probability and Random Processes. Oxford: Oxford University Press; 2001.

10. Galor O. Discrete Dynamical Systems. Berlin: Springer; 2007.

11. Diaconis P. Group representations in probability and statistics. Institute of Mathematical Statistics Lecture Notes; 1988.
